# Loss of vitellogenin receptor function results in yolk depletion, virome expansion, and reduced bacterial load within the oocytes of *Rhodnius prolixus*

**DOI:** 10.1186/s12915-026-02622-7

**Published:** 2026-05-13

**Authors:** Juliana Amorim, Jéssica Pereira, Matheus das Neves, Thamara Rios, Cintia Lopes Nogueira, Valdir Braz, Leonan Reis, Allana Faria-Reis, Ana Beatriz Walter-Nuno, Lukas Selim, Daiene Nunes, Thalysson Vinícius de J. C. Baptista, Georgia C. Atella, Marinella Silva Laport, Ana C. Bahia, Carlos Logullo, Pedro L. Oliveira, Gabriela O. Paiva-Silva, Katia C. Gondim, Isabela Ramos

**Affiliations:** 1 Instituto de Bioquímica Médica Leopoldo de Meis, Universidade Federal Do Rio de Janeiro, Rio de Janeiro, Brazil; 2 Instituto de Microbiologia, Universidade Federal Do Rio de Janeiro, Rio de Janeiro, Brazil; 3 Instituto de Biofísica Carlos Chagas Filho, Universidade Federal Do Rio de Janeiro, Rio de Janeiro, Brazil

**Keywords:** Vitellogenin receptor, Yolk uptake, Oogenesis, Vertical transmission, Microbiota homeostasis, Chagas disease vector

## Abstract

**Background:**

The vitellogenin receptor (VgR) mediates yolk protein uptake during follicle development and is essential for embryogenesis in oviparous species. Here, we characterize the single *Rhodnius prolixus VgR* isoform and uncover an unexpected role in microbial regulation within the reproductive system.

**Results:**

The receptor displays a conserved LDLR-like structure and is highly expressed in early oocytes. RNAi-mediated *VgR* silencing caused defective yolk granule biogenesis, leading to the accumulation of the main yolk protein precursors, Vg and RHBP, in the hemolymph, yet oviposition and fertilization proceeded normally. The resulting eggs were yolk-depleted and non-viable. Remarkably, *VgR* knockdown reduced bacterial 16S rRNA levels in oocytes while promoting the expansion of several members of the core virome, a phenotype not reproduced by Vg silencing. Neither purified Vg nor changes in immune (defensin) or RNA interference pathways explained the microbial shifts.

**Conclusions:**

These findings indicate that VgR influences not only yolk endocytosis but is also associated with changes in microbial composition within developing oocytes. We propose that VgR may contribute to a functional link between yolk endocytic dynamics and microbial homeostasis, thereby affecting the balance of microbial components in the oocyte. This connection broadens the functional context of VgR and provides new insight into how vertical transmission processes may be shaped in this major Chagas disease vector.

**Supplementary Information:**

The online version contains supplementary material available at 10.1186/s12915-026-02622-7.

## Background

*Rhodnius* *prolixus* is a hematophagous hemipteran that serves as one of the principal vectors of *Trypanosoma cruzi*, the causative agent of Chagas disease. This neglected tropical disease (NTD) is endemic to Central and South America, affecting approximately 8 million individuals, with significant morbidity [1]. Additionally, *R. prolixus* holds historical importance as a foundational model organism in studies of insect physiology, endocrinology, and development, with its utility enhanced by the availability of its genome [2]. Consequently, *R. prolixus* remains a dual-focus species of both medical significance and scientific inquiry, enabling advances in molecular and physiological research with implications for vector management [3].

To assure a sufficient supply of nucleic acids, proteins, lipids, phosphate, carbohydrates, ions, and vitamins required for the future embryo’s autonomous growth, developing oocytes in oviparous species, including insects, rely on the accumulation of large volumes of yolk. In most insects, the majority of the components of the yolk are produced extraovarially in the fat body, secreted into the hemolymph and carried into the developing oocytes by membrane-bound receptors via receptor-mediated endocytosis [4, 5]. Vitellogenin (Vg) is by far the most prevalent yolk protein in insect species [6]. Vg Receptors (VgR) are members of the low-density lipoprotein receptor (LDLR) family and mediate the accumulation of Vgs into the oocytes of many insects and vertebrate species [7]. Following binding, Vg/VgR complexes follow a canonical endocytic route leading to the formation of early and late endosomes, with recycling of VgR to the oocyte’s membrane. The endocytic route ends in the formation of mature membrane-bound organelles, named yolk granules (YG), where Vg is further processed and stored as vitellin (Vt) for later use [4, 8]. Typically, Vt and its products of degradation are the main source of nutrients that supply the embryo with fundamental molecules for de novo protein synthesis until the newly hatched first instar nymphs become capable of feeding [9].

Insect VgRs are distinct due to possessing two clusters of LBD (ligand-binding domain) and EGFD (epidermal growth factor-like domain), hence forming a different subclass within the LDLR family of receptors. The variations in the quantity and configuration of these structural units are believed to account for the recognition of the many ligands that are associated with this receptor family [7]. Across insects, VgR mRNA is generally expressed throughout oogenesis often peaking in previtellogenic or vitellogenic stages. In situ and immunolocalization studies show that VgR is evenly distributed in the oocyte during previtellogenesis and later accumulates at the oocyte cortex during vitellogenesis, consistent with its role in mediating yolk uptake [10–13]. Functional studies across diverse arthropod species have consistently demonstrated that RNAi-mediated silencing of the *VgR* invariably disrupts oogenesis and female fertility. Collectively, these findings underscore the conserved and essential role of VgR in insect reproduction [14–22].

In *R. prolixus*, transcriptomic analyses detected *VgR* transcripts in both the fat body and the ovaries at day 3 after blood feeding, with expression levels approximately 700-fold higher in the ovaries than in the fat body, indicating substantial ovarian accumulation of *VgR* mRNA. In the same datasets, *VgR* was found to be upregulated in the ovaries of unfed females, despite Vg uptake occurring only after a blood meal. Given the asynchronous development of ovarioles in triatomines and the continuous requirement for yolk uptake during the vitellogenic period, it has been proposed that elevated *VgR* transcript levels in the previtellogenic state may function to stockpile mRNA, enabling rapid translation and efficient receptor-mediated endocytosis following blood feeding [23].

Insects host a wide variety of microbial partners, ranging from mutualistic bacteria to pathogenic and symbiotic viruses, many of which have evolved mechanisms to persist and be transmitted across generations. Bacterial endosymbionts such as *Wolbachia*, *Rickettsia*, and *Spiroplasma* are ubiquitous among arthropod taxa and can profoundly influence host reproduction, immunity, and vector competence through vertical transmission via the germ line [24]. The triatomine *R. prolixus*, for instance, harbor the actinobacterium *Rhodococcus rhodnii* in its intestinal tract, a symbiont that provides essential nutrients for the insect’s development. This association is highly species-specific and critical for normal growth and fecundity of the host [25]. Similarly, vertically transmitted viruses are increasingly recognized as widespread and ecologically relevant components of insect microbiomes. Sigma viruses in *Drosophila* species, for instance, are inherited through both eggs and sperm and exemplify long-term host–virus coevolution [26]. Several plant and animal viruses exploit reproductive tissues for transmission. In the brown planthopper *Laodelphax striatellus*, the Rice stripe virus hijacks the VgR pathway to enter ovaries and achieve transovarial transmission [27, 28]. These intimate associations illustrate how reproductive molecular machinery can serve as a gateway for microbial persistence in insect populations. Consistent with this, Brito et al. (2021) [29] recently described a complex core virome in *R. prolixus* oocytes, composed of seven RNA viruses (RpV1–7) that are vertically transmitted to the progeny, unveiling a stable and heritable viral community integrated into the reproductive biology of this major Chagas disease vector.

In this study, we characterized the single *VgR* isoform of *R. prolixus* and investigated its roles in reproduction, yolk accumulation, and microbial transfer to the oocytes. We show that the *R. prolixus* VgR is a structurally conserved member of the LDL receptor superfamily and is predominantly expressed in the ovary. Functional silencing of *VgR* by RNA interference (RNAi) did not affect blood digestion, reproductive endocrine signaling, or oviposition but resulted in defective yolk uptake, leading to the production of yolk-depleted eggs unable to sustain embryogenesis. These phenotypes were accompanied by the accumulation of the main yolk protein precursors, Vg and Rhodnius heme-binding protein (RHBP), in the hemolymph and by altered bacterial and viral loads in the oocytes, indicating that VgR-mediated endocytosis influences not only yolk deposition but also the transfer of microbial components to the developing oocytes. Together, our findings identify VgR as a key regulator of oocyte maturation in *R. prolixus* and reveal its potential involvement in the transovarial passage of microorganisms, highlighting a connection between yolk accumulation and vertical transmission of microbiota in triatomine.

## Results

### Rhodnius prolixus harbors a conserved, single-copy VgR transcript that is highly enriched in early-stage oocytes

**Fig. 1:**
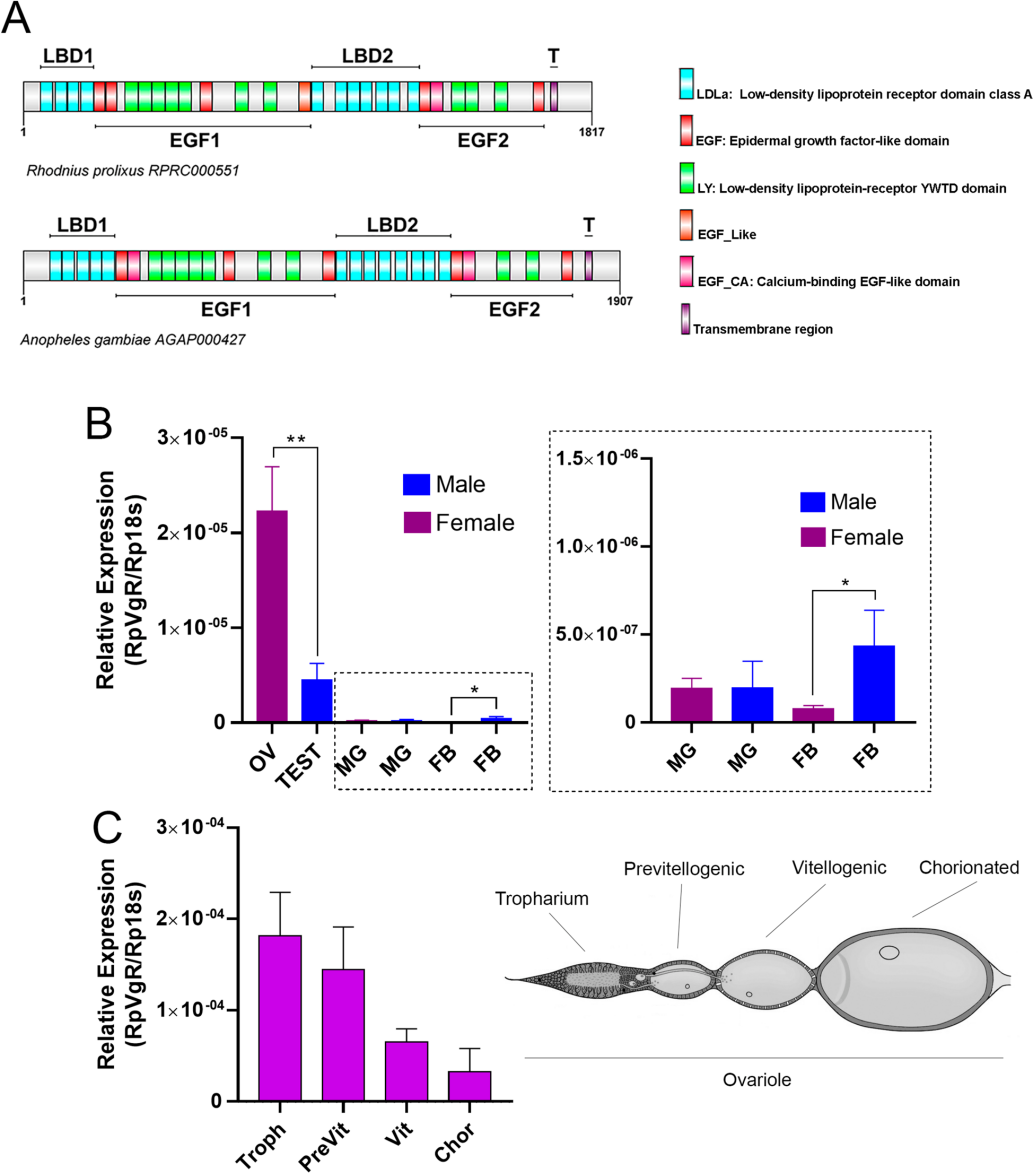
*Rhodnius prolixus* expresses a single conserved *VgR* isoform enriched in early oocytes. **A** Schematic diagram showing the conserved domain of *R. prolixus* and *Anopheles gambiae VgR*. The graphic was generated using the DOG 2.0 software (https://dog.biocuckoo.org). The conserved domains were identified using online software SMART. **B** RT-qPCR showing the expression profile of *VgR* in the different organs of adult males and females dissected 7 days after blood feeding. *VgR* expression is significantly higher in the ovary (OV) and testis (TEST) when compared to the fat body (FB) and midgut (MG) (*n* = 6–10). **C** RT-qPCR showing the expression profile of *VgR* during oocyte development. Troph, tropharium; PreVit, previtellogenic; Vit, vitellogenic; Chor, chorionated (*n* = 3–4) (*p* > 0.05). A schematic representation of the ovariole is shown. Graphs show mean ± SEM. *T*-test, one-way ANOVA. *p < 0.05, **p < 0.01

In *R. prolixus*, the sequence RPRC000551 was previously identified by [23, 30] as the single isoform of the *VgR* in this species. The *VgR* transcript comprises 5454 nucleotides, encoding a putative protein of 1817 amino acids. Domain analysis revealed that the predicted protein contains all conserved structural motifs typical of VgRs, including LDLa (low-density lipoprotein receptor class A; pfam00057), EGF (epidermal growth factor-like; pfam00008), LY (low-density lipoprotein receptor YWTD; pfam00005), EGF_like (pfam07974), and EGF_CA (calcium-binding EGF-like; pfam07645) domains, as well as a C-terminal transmembrane domain (Fig. 1A). We next quantified *VgR* transcript levels in different tissues of adult *R. prolixus* using RT-qPCR. Given the central roles of the midgut, fat body, and the ovary in the process of vitellogenesis and VgR-mediated yolk uptake, we focused our expression analyses on these three tissues. In adult females, *VgR* expression was two orders of magnitude higher in the ovary compared with the levels detected in the midgut and fat body. Interestingly, in adult males, approximately 25% of the expression observed in the female ovary was detected in the testis, indicating that *VgR* transcription is not restricted to females. Moreover, the male fat body expressed about five times more *VgR* than the female fat body (Fig. 1B). Within the ovary, *VgR* mRNA levels were 2–3 times higher in the tropharium and in fractions enriched in pre-vitellogenic follicles than in vitellogenic and chorionated (mature) oocytes (Fig. 1C). Together, these data indicate that *VgR* expression is predominantly female- and ovary-biased, but also present in male tissues, and dynamically regulated throughout oocyte development.

### Systemic VgR knockdown extends lifespan without disrupting vitellogenesis endocrine readouts

**Fig. 2:**
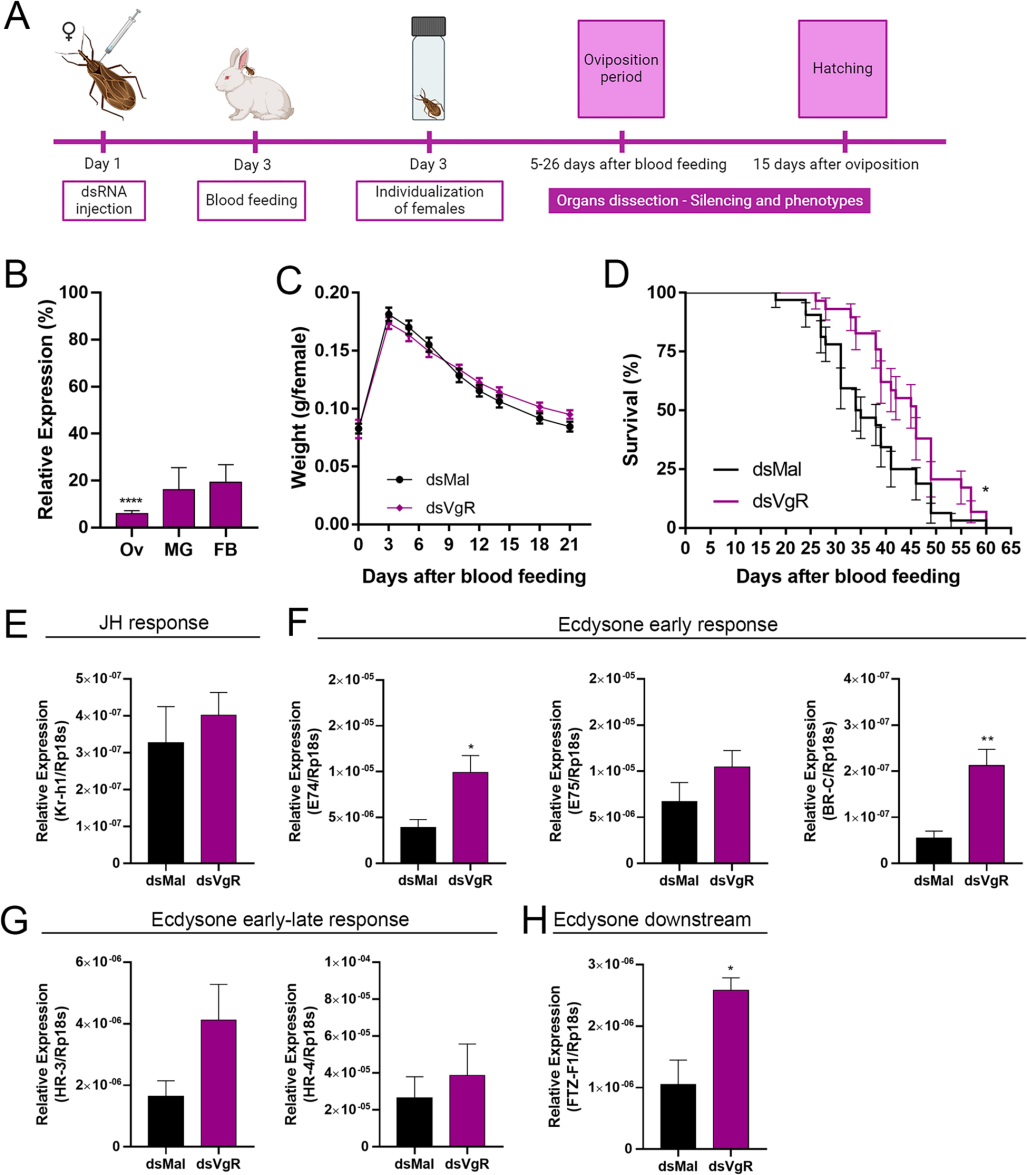
*VgR* knockdown extends female lifespan without impairing vitellogenic physiology. **A** Experimental design for dsRNA injection. Insects were injected 2 days before blood feeding, individualized, and the phenotypes were observed during the gonotrophic cycle. **B** Silencing efficiency 7 days after the blood meal is presented in different organs after injection of dsRNA designed to target *VgR* fat body (FB), midgut (MG), and ovary (OV) (*n* = 4–10). Values are relative to the dsMal control. *T*-test. **C** Effects of *VgR* silencing on female digestion/diuresis, showing their weight during the gonotrophic cycle (*n* = 30). Two-way ANOVA. *p* > 0.05. **D** Survival rates of control and *VgR*-silenced females (*n* = 30). Log-rank (Mantel-COX) test. **E** RT-qPCR showing the expression profile of the JH effector gene Kr-h1 in the ovary of control and *VgR*-silenced females 7 days after blood feeding (*n* = 5–6). *T*-test. *p* > 0.05.** F**–**H** RT-qPCR showing the expression profile of the ecdysone effector genes E74, E75, BR–C, HR3, HR-4, and FTZ-F1 in the ovaries of control and *VgR*-silenced females 7 days after blood feeding (*n* = 5–6). *T*-test. Graphs show mean ± SEM. *p < 0.05, ****p < 0.0001

To further explore the role of VgR in the physiology of adult females, we synthesized and injected dsRNAs targeting *VgR* 2 days before blood feeding and monitored the resulting phenotypes throughout the subsequent gonotrophic cycle (Fig. 2A). Injection of dsVgR achieved an efficient knockdown, with at least 80% reduction in *VgR* transcript levels across all analyzed organs (Fig. 2B). We first assessed whether *VgR* silencing affected key aspects of vitellogenic physiology. Insects were weighed immediately before blood feeding (day 0) and re-weighed 3 days after feeding. In *R. prolixus*, post-feeding weight loss strongly correlates with the progression of blood digestion [31–34]. Part of this reduction also reflects active diuresis, as insects eliminate excess ions and nitrogenous waste generated during blood processing [35]. Therefore, changes in body mass during the gonotrophic cycle provide an indirect measure of both digestion and excretory activity. No differences were observed in the rate of post-feeding weight loss between control and VgR-silenced females throughout the gonotrophic cycle (Fig. 2C), indicating that blood digestion and diuresis were not affected by VgR silencing. Interestingly, a significant increase in longevity was detected: the median survival increased from 34.5 days in control females to 46 days in *VgR*-silenced insects (Fig. 2D). To determine whether the reproductive phenotypes observed upon *VgR* silencing could be secondary to systemic endocrine imbalances, we measured representative effectors of the juvenile hormone (JH) and ecdysone signaling pathways, which are known regulators of vitellogenesis and oogenesis [36–40]. The JH-responsive gene Kr-h1 showed no significant changes following *VgR* knockdown (Fig. 2E). In contrast, some ecdysone-responsive genes, including E74, BR–C, HR-3, and FTZ-F1, were upregulated by at least twofold relative to controls, whereas E75 and HR-4 remained unaltered (Fig. 2F–H). These results indicate that *VgR* silencing does not impair the activation of primary reproductive endocrine pathways in adult female *R. prolixus*.

### VgR silencing blocks yolk uptake, causing hemolymph accumulation of Vg and RHBP

**Fig. 3:**
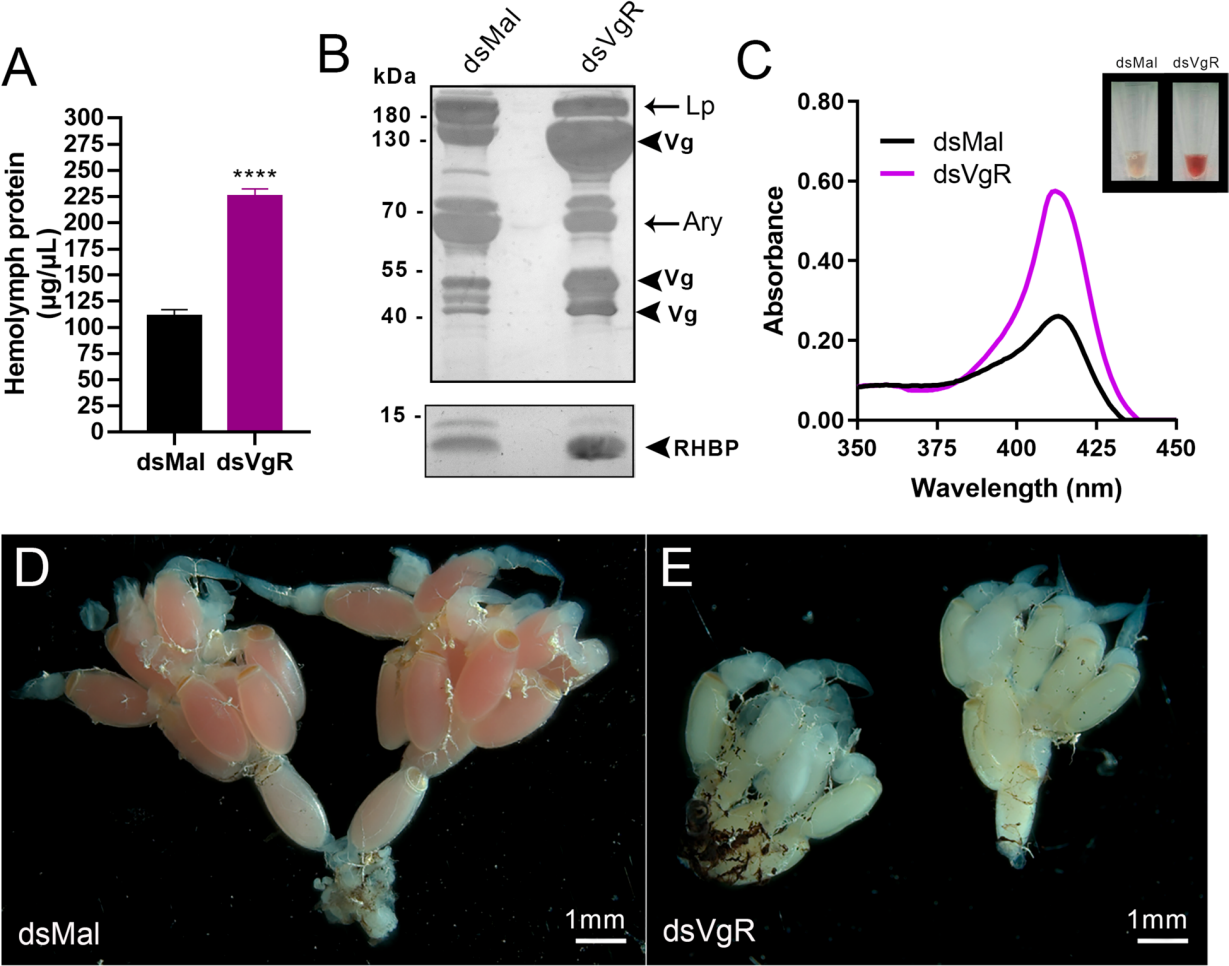
Impaired yolk uptake in *VgR*-silenced females leads to hemolymph accumulation of yolk proteins. **A** Control and silenced hemolymph protein quantifications 7 days after the blood meal (*n* = 6–10). *T*-test. **B** 10% (upper panel) and 13.5% (lower panel) SDS-PAGEs of the hemolymphs of control and *VgR*-silenced females 7 days after the blood meal (*n* = 6). Arrowheads point to the Vg subunits and RHBP. **C** Light absorption spectra of hemolymph from females injected with dsMal or dsVgR. RHBP Soret peak is shown at 412 nm for both conditions. Inset shows a representative image of the hemolymphs collected on day 7 after blood feeding. The results are representative of two independent experiments. **D**–**E** Representative images of ovaries dissected from control and *VgR*-silenced females 7 days after blood feeding. Scale bar: 1 mm. All graphs show mean ± SEM. ****p < 0.0001

To further investigate the effects of *VgR* silencing, we quantified the total protein content in the hemolymph of vitellogenic females. *VgR*-silenced females contained approximately twice as much total protein as control insects (Fig. 3A). Analysis of hemolymph protein composition by SDS-PAGE revealed a marked accumulation of vitellogenin (Vg) [41] in *VgR*-silenced females. This accumulation was accompanied by an increase in RHBP [42, 43], but not in the other major hemolymph proteins, lipophorin (Lp) [44, 45] and arylphorin (Ary) [46] (Fig. 3B, Additional File 1: Fig. S1). Densitometric analysis revealed that Ary levels were significantly reduced (~ 40%) in VgR-silenced insects, while Lp showed no significant change (Fig. 3B, Additional File 1: Fig. S2). Consistent with these findings, the hemolymph of *VgR*-silenced females exhibited a reddish coloration, characteristic of RHBP accumulation (Fig. 3C, inset) [42, 43, 47, 48]. The titration of total RHBP in the hemolymph was determined following the protocol described by [49]. Hemolymph samples were saturated by the addition of hemin, and absorbance spectra were recorded. The characteristic Soret peak at 412 nm confirmed the higher concentration of RHBP in dsVgR hemolymphs (Fig. 3C). In dsMal hemolymphs, the titration profile indicated concentrations of 201.1 ± 89.9 µM RHBP. In dsVgR, the profile indicated a higher total amount of RHBP, reaching 403.1 ± 98.6 µM. Upon dissection, the ovaries of *VgR*-silenced females displayed an altered morphology, characterized by smaller, pale (white) oocytes (Fig. 3E), in contrast to the reddish, mature oocytes observed in control insects (Fig. 3D). The absence of the red pigment indicates the lack of RHBP, which is known for being the one red molecule from the oocytes in *R. prolixus* [42, 47, 49]. Together, these results indicate that *VgR* silencing does not affect Vg synthesis or secretion by the fat body but rather impairs the uptake of both Vg and RHBP by developing oocytes, demonstrating that RHBP internalization is functionally linked to Vg endocytosis, and consequently leads to their accumulation in the hemolymph.

### Oviposition rates persist after VgR knockdown, but yolk-depleted eggs fail to support embryogenesis

**Fig. 4:**
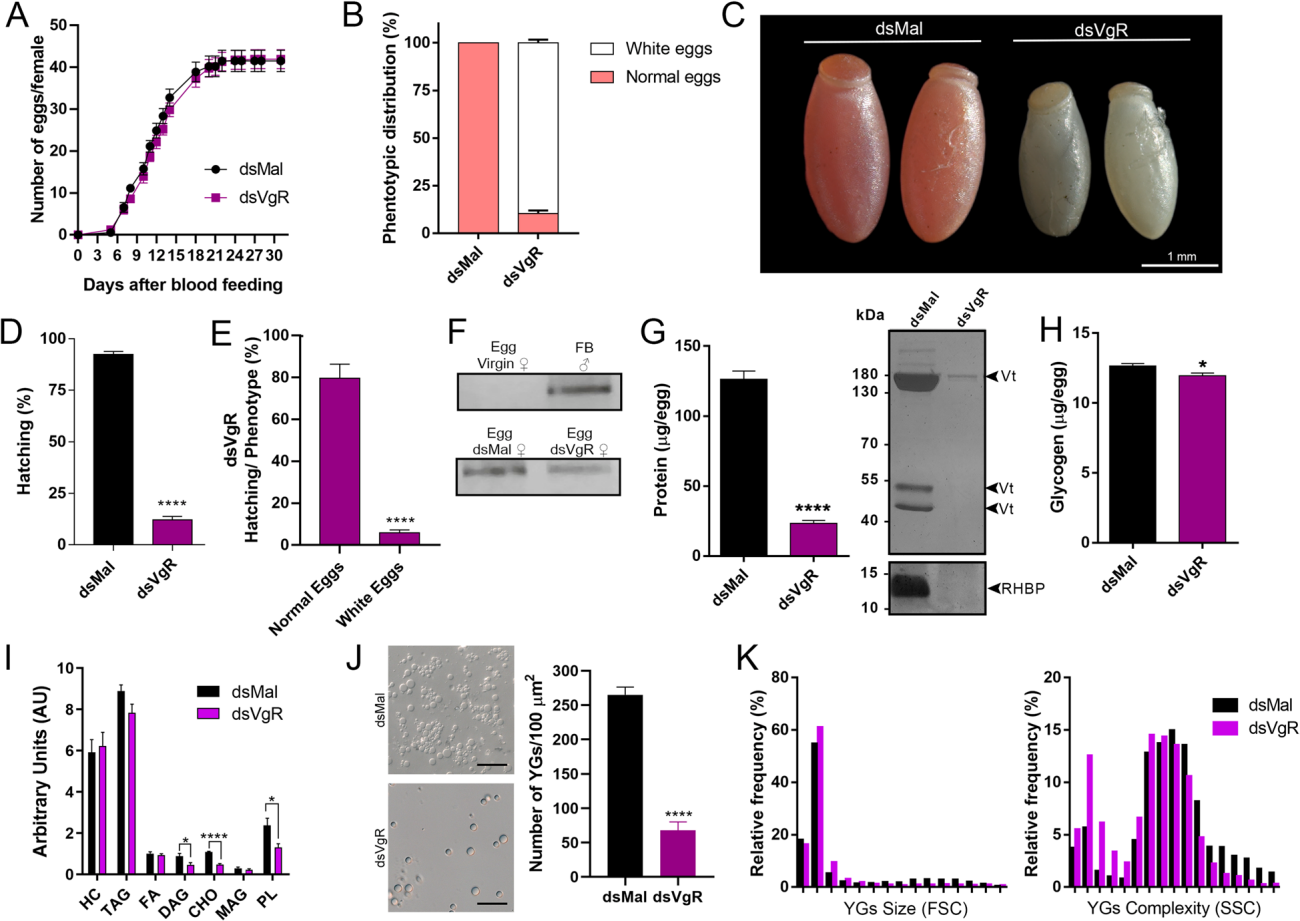
Yolk-depleted eggs from *VgR*-silenced females are fertilized but non-viable. **A** Oviposition rates control and silenced females during the gonotrophic cycle (*n* = 30). Two-way ANOVA. *p* > 0.05.** B** Phenotypic distribution of the eggs laid by control and *VgR*-silenced insects (*n* = 30). **C** Representative images of freshly laid eggs. Scale bar: 1 mm.** D** F1 total hatching rates after *VgR* silencing (*n* = 30). *T*-test. **E** F1 hatching rates per phenotype (*n* = 30). *T*-test.** F** Genomic DNA from eggs laid by control and *VgR*-silenced females were extracted and used as templates for amplifying a specific Y chromosome fragment by PCR. Genomic DNA from nonfertilized eggs laid by virgin females was used as negative controls. Fat body (FB) DNA from adult male insects was used as a positive control. **G** Protein levels of freshly laid eggs from control and *VgR*-silenced females (*n* = 4–7). *T*-test. SDS-PAGE protein profile of freshly laid eggs from control and *VgR*-silenced females (*n* = 4–7). Arrowheads point to Vitellin (Vt) and RHBP. **H** Glycogen content quantified in eggs laid by control and VgR females (*n* = 7). *T*-test. **I** Lipid analysis was performed by TLC. Diacylglycerol (DAG), cholesterol (CHO), phospholipids (PL), hydrocarbons (HC), triacylglycerol (TAG), monoacylglycerol (MAG) (*n* = 8). *T*-test. **J** Representative images of the yolk granules (YGs) extracted from control and *VgR*-silenced females observed under the light microscope (*n* = 2). Scale bar: 30 μm. Quantifications of the number of YGs extracted from dsMal and dsVgR oocytes (*n* = 5). *T*-test. **K** The YGs suspensions were analyzed by flow cytometry. Frequency histogram analysis reveals a shift in the size (FSC) and complexity (SSC) of YGs in eggs laid by *VgR*-silenced females (*n* = 16–22). All Graphs show mean ± SEM. *p < 0.05, ****p < 0.0001

Despite the abnormal morphology of the oocytes, *VgR*-silenced females oviposited normally, with no significant differences in oviposition rates compared to control insects (Fig. 4A). However, examination of the eggs laid by *VgR*-silenced females revealed severe phenotypic alterations: only 5% of the eggs laid by *VgR*-silenced females displayed normal morphology, while 95% were small and white (Fig. 4B–C). Overall, these eggs exhibited low viability, with a total hatching rate of approximately 10% (Fig. 4D). Among morphologically normal eggs, 79% of embryos were viable, whereas only 4% of embryos from the white eggs were successfully developed (Fig. 4E). Despite their reduced size and lack of pigmentation, the white eggs were fertilized, as confirmed by PCR detection of a Y chromosome transcript, following the method previously described by [49] (Fig. 4F). Biochemical analyses revealed that eggs from *VgR*-silenced females exhibited an ~ 80% reduction in total protein content, accompanied by drastically reduced levels of vitellin (Vt) and RHBP (Fig. 4G, Additional File 1: Fig. S3). In contrast, despite the smaller egg volume, the total glycogen content of these eggs was only slightly reduced when compared to controls (Fig. 4H). Lipid analysis by thin-layer chromatography (TLC) showed that hydrocarbon (HC), triacylglycerol (TAG), fatty acid (FA), and monoacylglycerol (MAG) showed similar levels in control and silenced groups (Fig. 4I). However, the contents of diacylglycerol (DAG), cholesterol (CHO), and phospholipid (PL) were reduced by approximately 55%, 68%, and 45%, respectively (Fig. 4I, Additional File 1: Fig. S4). Importantly, although total lipids were not independently quantified as a separate biochemical measurement, the densitometric analyses presented reflect absolute values for each lipid class. Thus, the sum of all quantified lipid classes corresponds to total lipid content, indicating an overall reduction in total lipids driven specifically by decreases in DAG, CHO, and PL, rather than by a uniform decline across all lipid categories. Together, these findings demonstrate that *VgR* silencing does not impair oviposition or fertilization, but severely disrupts yolk deposition, resulting in protein- and lipid-depleted eggs unable to sustain embryonic development. Because CHO and PL are major components of biological membranes, their reduction in *VgR*-silenced eggs, together with the observed yolk depletion, led us to hypothesize that yolk granule (YG) biogenesis was compromised. To test this, YG suspensions were prepared by directly disrupting the eggs on a glass slide and examining them under differential interference contrast (DIC) microscopy. We found that the number of YGs was reduced by approximately 70% in oocytes from *VgR*-silenced females compared to controls (Fig. 4J). To further characterize the YG population, we used flow cytometry to analyze YGs isolated from chorionated oocytes of control and *VgR*-silenced females, as previously described by [50, 51]. The analysis provided information on YG size (forward scatter, FSC) and internal complexity (side scatter, SSC). The resulting frequency histograms (Fig. 4K) revealed that, although YGs from both groups displayed a wide range of sizes and complexities, those from *VgR*-silenced oocytes were predominantly smaller and less complex than those from controls. Together, these findings demonstrate that loss of VgR function impairs YG formation, resulting in defective YG biogenesis and supporting the idea that VgR-mediated endocytosis is essential for yolk accumulation during oocyte maturation.

Although VgR is classically associated with female reproduction, it has been reported in males of other species [22, 52–54]. Consistently, we detected VgR expression in adult male tissues, with testis levels reaching approximately 20% of those observed in female ovaries (Fig. 1B, Additional File 1: Fig. S5A). To investigate possible male functions, we systemically silenced *VgR* in adult males using the same dsRNA protocol applied to females. Knockdown efficiency reached at least 65% in fat body, midgut, and testis (Additional File 1: Fig. S5B), without affecting blood digestion (Additional File 1: Fig. S5C) or male longevity (Additional File 1: Fig. S5D). Because Vg silencing in male *Chrysopa pallens* reduces oviposition and hatching in mated females [55], we tested whether *VgR* silencing in male *R. prolixus* produced similar effects. *VgR*-silenced males were mated with wild-type virgin females (Additional File 1: Fig. S5E), and knockdown efficiency in the testis at the time when males were paired with the females reached at least 85% (Additional File 1: Fig. S5F), confirming that the males used in the mating assays were effectively silenced. However, neither female oviposition (Additional File 1: Fig. S5G) nor egg hatching rates (Additional File 1: Fig. S5H) were affected. Together, these findings indicate that although *VgR* is expressed in males, its silencing does not impair male physiology, fertility, or offspring development in *R. prolixus*.

### VgR silencing reshapes oocyte bacterial and core virome loads, and these effects are not explained by changes in Vg abundance in the oocyte or the hemolymph

**Fig. 5:**
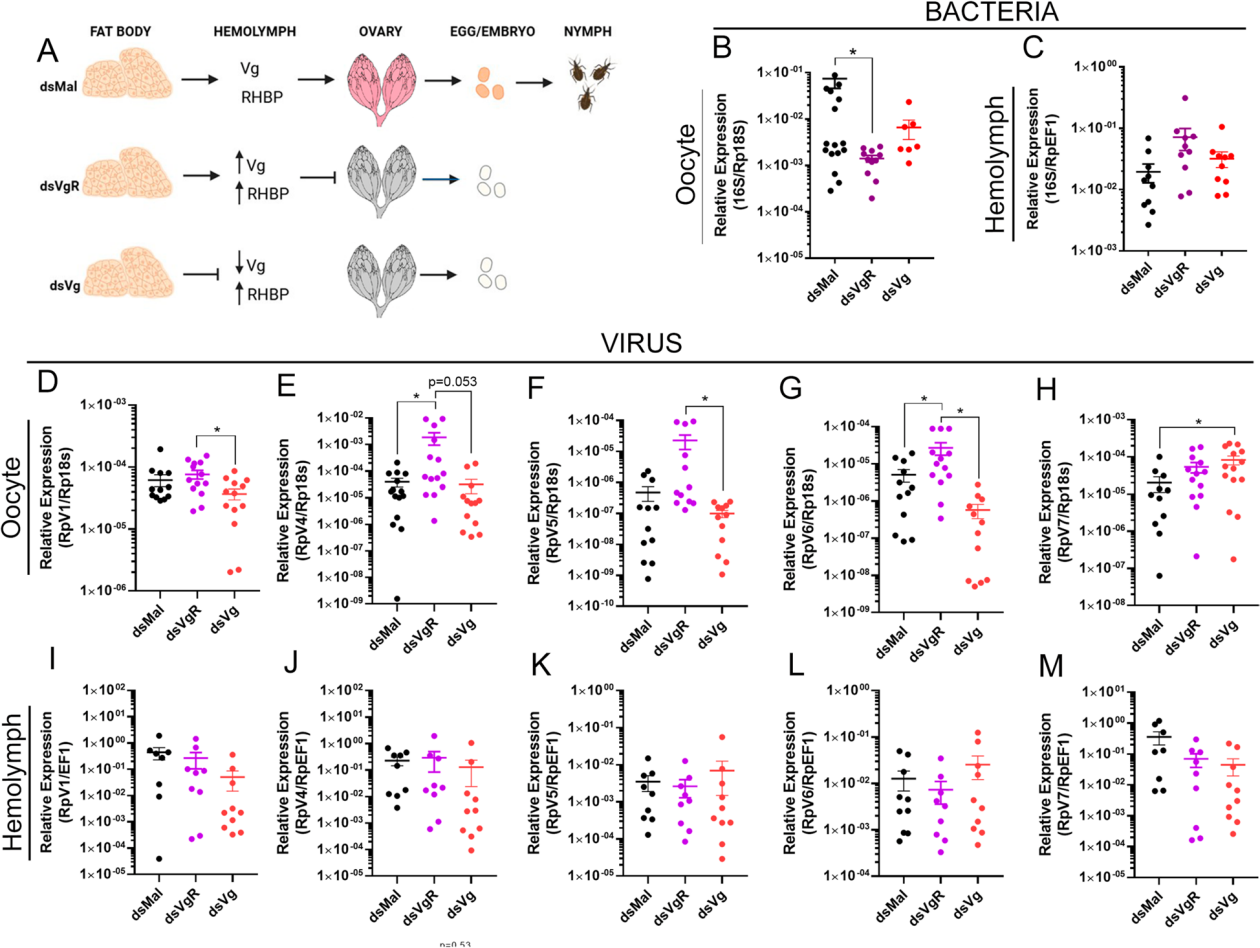
*VgR* knockdown reshapes bacterial and viral composition of oocytes.** A** Schematic representation of hemolymph, ovary, and embryo phenotypes following *VgR* or *Vg* silencing in *R. prolixus*. *Vg* data are from Pereira et al. (2025). *VgR* silencing leads to accumulation of Vg in the hemolymph, yolk-depleted eggs, and unviable embryos. *Vg* silencing results in Vg-depleted hemolymph, yolk-depleted eggs, and unviable embryos. In both cases, RHBP levels are increased in the hemolymph. **B** RT-qPCR analysis of bacterial 16S rRNA levels in oocytes from *VgR*- and *Vg*-silenced females (*n* = 7–16). **C** RT-qPCR analysis of bacterial 16S rRNA levels in the hemolymph (*n* = 10). **D**–**H** RT-qPCR quantification of different viral components of the core oocyte virome of *R. prolixus* in oocytes from *VgR*- and *Vg*-silenced females (*n* = 12–13). **I**–**M** RT-qPCR quantification of the same viral components in the hemolymph (*n* = 10). All graphs show mean ± SEM. One-way ANOVA; *p < 0.05

Because yolk accumulation is known to participate in the shuttling of microbes to the oocyte and contributes to the vertical transmission of both pathogenic and non-pathogenic microbiota [27, 28, 56–60], we investigated bacterial and viral levels in yolk-depleted oocytes. In addition to *VgR* silencing, direct *Vg* silencing also produces yolk-depleted eggs that are morphologically and biochemically similar to those observed under *VgR* knockdown [61]. However, the physiological context differs markedly between the two conditions. As shown in Fig. 5A, *VgR* silencing results in high circulating Vg, whereas *Vg* silencing leads to Vg-depleted hemolymph [61]. Because the hemolymph is likely the main route through which microbes reach the oocytes, we also asked whether bacterial and viral loads within the oocytes would reflect differences in Vg abundance in the hemolymph. To address this, we quantified bacterial and viral levels in both oocytes and hemolymph from *Vg*- and *VgR*-silenced insects (Fig. 5). In oocytes from *VgR*-silenced insects, qPCR analysis of the bacterial 16S rRNA gene revealed a marked reduction in bacterial load (Fig. 5B). Regarding the *R. prolixus* oocyte core virome, seven viruses were initially described [29]. In our qPCR analyses, RpV3 transcript levels were consistently near the detection threshold and were therefore excluded from further analyses. RpV2, although reported in the original transcriptomic dataset, was not reliably detected by qPCR either in our assays or in the validation experiments of the foundational study and has been speculated to have been lost from the colony over time [29]. Among the remaining five detectable viruses, VgR-silenced oocytes displayed increased levels of RpV4, RpV5, RpV6, and RpV7 (Fig. 5D–H). In contrast, the reduction in bacterial load was less pronounced in Vg-silenced eggs compared with those lacking VgR (Fig. 5B). For the viruses, all members except RpV7 tended to decrease in Vg-silenced oocytes (Fig. 5D–H). Since both *Vg-* and *VgR-*silencing result in morphologically and biochemically similar yolk-depleted eggs, we hypothesized that the observed differences in bacterial and viral levels between *Vg*- and *VgR*-silenced females might reflect alterations occurring in the hemolymph. To test this possibility, we quantified bacterial and viral loads in the hemolymphs of both conditions. Hemolymph samples exhibited more dispersed bacterial (Fig. 5C) and viral levels (Fig. 5I–M), without consistent trends correlating with those observed in the oocytes.

### Bacterial and viral load changes are not explained by direct Vg antimicrobial activity

Previous studies have shown that purified Vg from species such as *Apis mellifera*, *Panulirus longicornis*, *Cyprinus carpio*, and *Bradyrhizobium japonicum* were experimentally tested against bacterial strains, typically *Escherichia coli* and/or *Staphylococcus aureus*, showing direct antibacterial activity (e.g., growth inhibition, membrane disruption, or bacteriostatic effects) [60]. To test whether *R. prolixus* Vg displays antibacterial activity, purified Vg was incubated with *E. coli* and *S. aureus* cultures for 24 h at 37 °C. Bacterial growth was monitored and relative growth was calculated against untreated controls. Across all concentrations tested (12.5–200 µg/mL), *E. coli* growth remained between 91 and 99% of control levels (Additional File 1: Table S2), while *S. aureus* growth ranged from 99 to 108% of controls (Additional File 1: Table S3). No concentration-dependent inhibition was observed for either bacterial species, indicating that purified *R. prolixus* Vg does not exert detectable bacteriostatic or bactericidal effects under these conditions.

Viral infectivity was evaluated using a standard plaque assay for Mayaro virus (MAYV), and the number of plaque-forming units (PFU/mL) was quantified to determine viral titers. No significant differences in plaque number or morphology were observed between control and Vg-treated (400 µg/mL) groups, with similar PFUs for both conditions, indicating that Vg treatment did not alter MAYV infectivity or replication efficiency under the tested conditions (Additional File 1: Fig. S6). Altogether, the data indicates that the observed alterations in viral and bacterial loads are unlikely to result from the direct action of Vg within the oocytes.

### Ovaries are immunocompetent, but defensin and RNAi pathway modulation do not explain microbial shifts in VgR knockdown oocytes

**Fig. 6:**
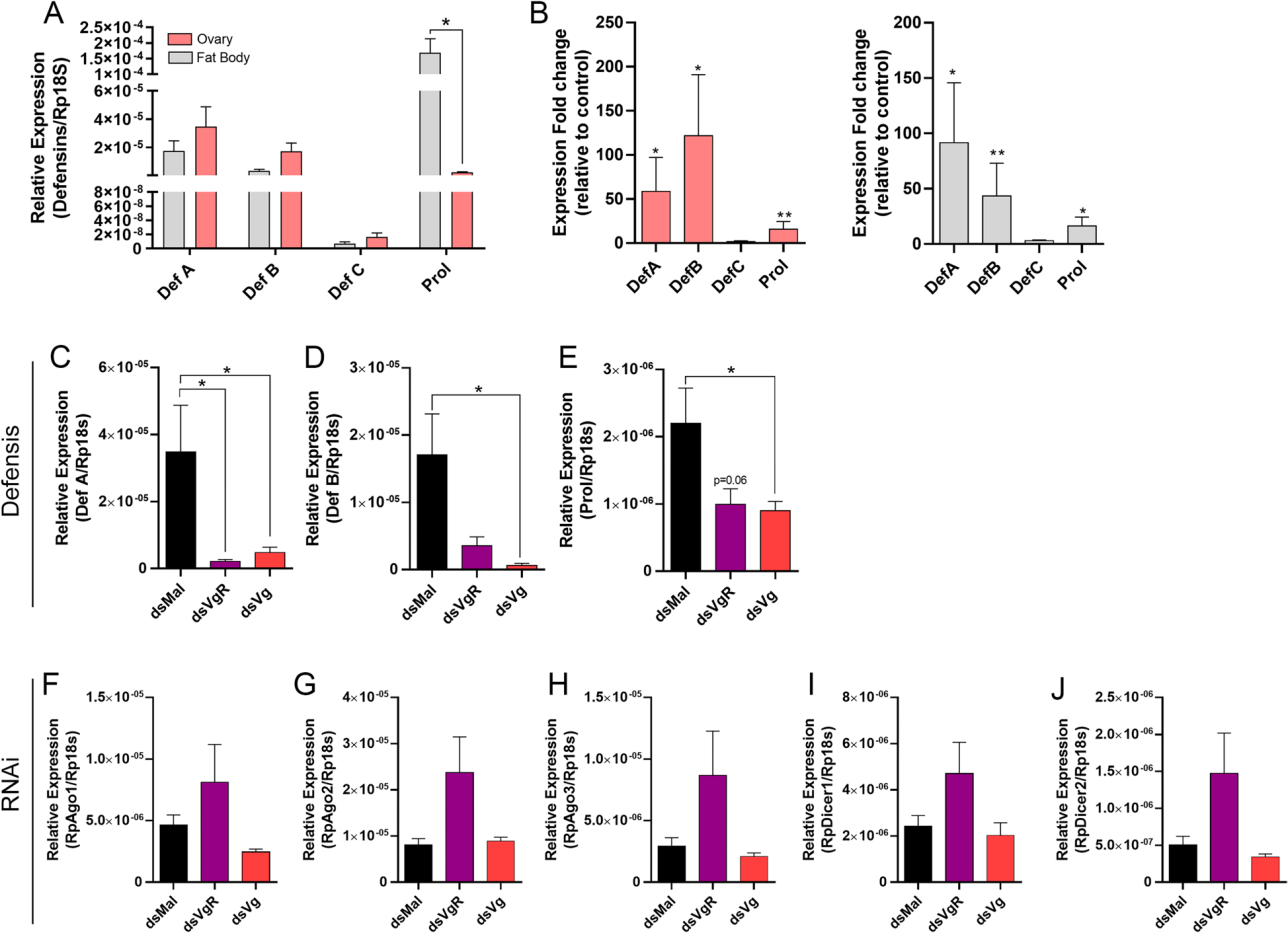
Ovary immune responses are present, but defensin and RNAi modulation do not explain microbial shifts.** A** RT-qPCR showing the basal expression levels of *Defensin A*, *Defensin B*, *Defensin C*, and *Prolixicin* in the fat body and ovary of *R. prolixus* (*n* = 8–9). **B** Induction of the same antimicrobial peptide genes in the fat body and ovary after *T. rangeli* infection (*n* = 6). Fold-change values are expressed relative to unchallenged control insects. **C**–**E** Expression of *Defensin A*, *Defensin B*, and *Prolixicin* in the ovaries of *VgR*- and *Vg*-silenced females (*n* = 8–9). **F**–**J** Expression of core RNA interference pathway components (*Dicer-1*, *Dicer-2*, *Argonaute-1*, *Argonaute-2*, and *Argonaute-3*) in the ovaries of *VgR*- and *Vg*-silenced females (*n* = 8–9) (*p* > 0.05). All graphs show mean ± SEM. One-way ANOVA; *p < 0.05

Another possibility we considered to explain the observed microbial changes was that *VgR* silencing might influence the insect’s immune response, either systemically or within the ovary itself. Previous studies in *R. prolixus* have demonstrated that four antimicrobial peptides (*Defensin A*,* B*, and *C*, and *Prolixicin*) are upregulated in the midgut and fat body following immune challenges with *T. cruzi*, *Trypanosoma rangeli*, and bacteria [62–66]. However, whether the ovary also displays immune competence, that is, the ability to upregulate antimicrobial peptides in response to immune challenge, had not yet been investigated. To address this, we challenged insects with *T. rangeli*, a well-characterized pathogen in *R. prolixus* that systemically infects the vector and is known to robustly induce AMP expression, thereby providing a reliable model to assess tissue-specific immune responsiveness [67–71]. We first assessed whether the ovary of *R. prolixus* is immunocompetent. Under pathogen-unchallenged conditions, qPCR revealed that the expression of *Defensin A* and *Defensin B* were approximately 25% and 80% higher, respectively, in the ovary than in the fat body, whereas *Prolixicin* exhibited a basal expression roughly tenfold greater in the fat body compared to the ovary. *Defensin C* exhibited approximately twofold higher expression in the ovary compared to the fat body; however, its overall transcript levels were at least 3 orders of magnitude lower than those of the other antimicrobial peptides (Fig. 6A). To evaluate immune activation, we analyzed the response of these antimicrobial peptides to infections with *T. rangeli*, using a protocol previously described by [72]. Both the fat body and the ovary upregulated all four *defensin* genes upon infection, reaching comparable induction levels in both tissues (Fig. 6B). These results demonstrate that, in addition to the fat body, the ovary of *R. prolixus* is an immune-responsive tissue capable of mounting a transcriptional response to immune challenge.

Having established that the ovary is immunocompetent, we next examined *defensin* expression in *VgR*- and *Vg*-silenced females to determine whether the observed changes in bacterial loads were associated with altered immune response. In both knockdowns, the expression levels of all *defensin* genes were markedly reduced in the ovary (Fig. 6C–E), a pattern that correlated negatively with the decreased bacterial load detected by 16S rRNA qPCR. Thus, modulation of canonical immune effectors is unlikely to account for the differences in bacterial abundance observed in the oocytes of *VgR*- and *Vg*-silenced insects.

Brito et al. (2021) [29] demonstrated that RNAi represents the primary mechanism controlling the expansion of the *R. prolixus* core virome during oogenesis. In that study, the authors detected abundant 22-nucleotide viral small interfering RNAs (vsiRNAs) targeting all members of the core virome and proposed that vsiRNA-mediated silencing is likely the main mechanism regulating viral load in the oocytes. In this system, viral double-stranded RNA intermediates are processed by Dicer into vsiRNAs, which are loaded into Argonaute-containing RISC complexes, where Argonaute acts as the catalytic slicer that cleaves complementary viral RNAs to suppress replication. To further investigate the basis of the viral load alterations observed in our knockdown oocytes, we examined the expression of key RNAi components to determine whether this pathway might be modulated in *VgR*- and *Vg*-silenced females. Interestingly, VgR silencing, but not Vg silencing, was associated with a tendency toward upregulation of all Argonaute and Dicer isoforms; however, these changes were not statistically significant (Fig. 6F–J), concomitant with the increased viral loads detected in these oocytes. Thus, a modulation in the RNAi pathway does not appear to be the cause of the observed differences in viral abundance, suggesting that other regulatory mechanisms may influence virome dynamics in *VgR*- and *Vg*-silenced ovaries.

### VgR is not transcriptionally modulated in Vg-silenced oocytes

**Fig. 7:**
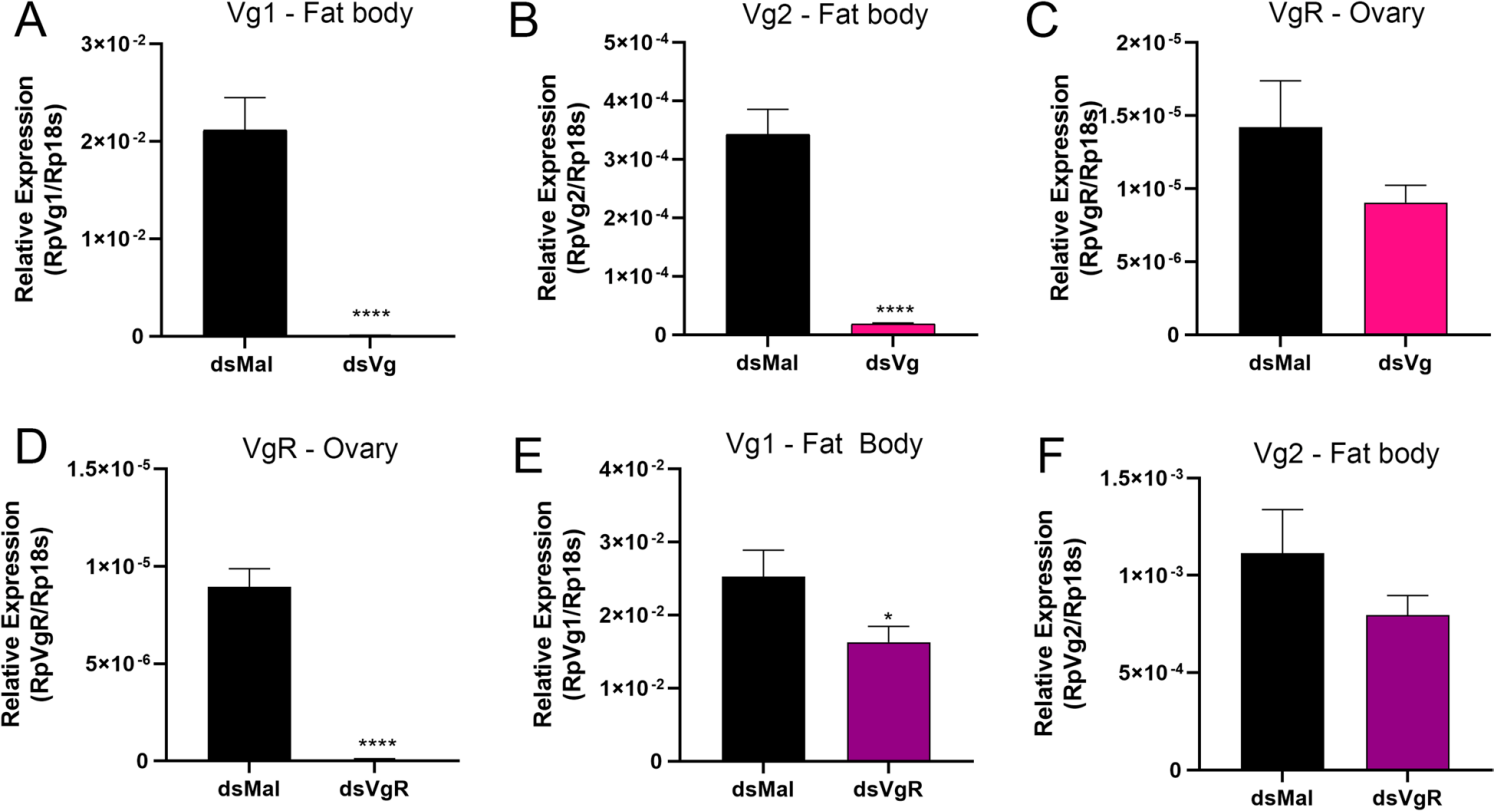
*VgR* is not transcriptionally modulated in *Vg*-silenced oocytes.** A**–**B** RT-qPCR quantification of *Vg1* and *Vg2* transcript levels in the fat bodies of Vg-silenced females (*n* = 8–9). **C** *VgR* transcript levels in the ovaries of *Vg*-silenced females (*n* = 8–9). **D** RT-qPCR quantification of *VgR* transcript levels in the ovaries of *VgR*-silenced females (*n* = 8–9). **E**–**F** RT-qPCR quantification of *Vg1* and *Vg2* transcript levels in the fat body of *VgR*-silenced females (*n* = 8–9). Graphs show mean ± SEM. *T*-test; *p < 0.05. ****p < 0.0001

Next, we hypothesized that the opposite patterns of viral abundance observed between *VgR*- and *Vg*-silenced oocytes could be related to the differential presence of *VgR*. Specifically, we reasoned that oocytes from *Vg*-silenced females might upregulate *VgR* expression to compensate for the reduced availability of circulating Vg. In this scenario, the lower viral loads detected in *Vg*-silenced oocytes would result from increased *VgR* expression. To test this hypothesis, we quantified *VgR* transcript levels in oocytes from *Vg*-silenced females. The silencing efficiencies of *Vg1* and *Vg2* reached up to 90% in the fat body (Fig. 7A–B). However, *VgR* expression in the ovaries remained comparable to control samples (Fig. 7C), indicating that *VgR* transcript is not upregulated under *Vg*-deficient conditions and is therefore unlikely to be associated with the *Vg*-specific changes observed in bacterial and viral loads.

Since we assessed potential compensatory regulation of *VgR* in *Vg*-silenced insects, we also examined the reciprocal possibility, i.e., whether Vg genes would be counter-regulated in *VgR*-silenced females. This analysis was performed to determine whether any transcriptional feedback exists between these components. Although *VgR* was silenced by 95% in the ovaries (Fig. 7D), *Vg1* and *Vg2* transcript levels showed only a very modest tendency toward downregulation (Fig. 7E–F). These results indicate that VgR and Vg genes do not transcriptionally regulate each other, and that the accumulation of Vg in the hemolymph (Fig. 3) is not associated with increased *Vg* transcription but is likely largely attributable to impaired ovarian uptake of Vg proteins.

## Discussion

Vector-borne NTDs remain a major global health challenge, with Chagas disease affecting millions across Latin America and emerging globally [73, 74]. *R. prolixus*, a key vector and a classical model in insect physiology [3], offers some experimental advantages, including a fully sequenced genome [2] and extensive transcriptomic resources for digestive and reproductive tissues [23, 75–78]. Its robust RNAi response further supports its use in dissecting reproductive pathways and identifying molecular targets to reduce vector fertility.

In this context, *R. prolixus* expresses a single, structurally conserved *VgR* that is predominantly localized to the ovary, consistent with previous descriptions in other insects [7]. The high expression of *VgR* in early oocytes supports its role in receptor-mediated yolk uptake during vitellogenesis. Interestingly, *VgR* transcripts were also detected in the testis, suggesting that this receptor may have additional, yet unexplored, functions in male physiology, as also observed in other insect species [22, 52–54].

Beyond its reproductive effects, VgR silencing increased adult female longevity following *VgR* silencing. Vg has been implicated in lifespan regulation in several insects, most notably in *A. mellifera*, where high Vg levels are associated with extended lifespan in long-lived winter workers and correlate inversely with JH levels [79]. In that system and other species, endocrine pathways such as Insulin/IGF signaling, JH, and ecdysteroids are tightly linked to both reproduction and aging [79–84]. In *R. prolixus*, silencing of Vg [61] and of the insulin receptor (InsR) [34] also extends lifespan, supporting the idea that endocrine and metabolic networks influence longevity in this species. Interestingly, however, Vg knockdown extended lifespan in both females and males, whereas in the present study *VgR* silencing increased lifespan only in females. This discrepancy challenges the assumption that the mechanisms linking altered Vg/VgR pathway to longevity are equivalent, suggesting that these processes may influence lifespan through partially distinct pathways. Importantly, while reduced reproductive investment is often proposed to extend lifespan, the mechanisms underlying this relationship remain debated [85]. Thus, altogether, our findings reinforce that the interplay between Vg/VgR function, endocrine regulation, and longevity in insects is complex and multifactorial and will require further investigation in *R. prolixus*.

The altered protein profile in the hemolymph upon VgR silencing reinforces that VgR plays a central role in the coordinated uptake of major yolk components by the oocyte. The marked accumulation of Vg and RHBP, together with the increased total protein content, indicates that their synthesis and secretion by the fat body remain active, but their internalization is impaired. The concomitant reduction in Ary suggests that VgR silencing may also indirectly affect the systemic balance of hemolymph proteins, possibly through compensatory or metabolic adjustments. Importantly, the absence of RHBP in the oocytes, reflected by their pale phenotype, highlights that RHBP uptake is functionally coupled to Vg endocytosis. Together, these findings support a model in which VgR-mediated trafficking is not limited to Vg alone but is part of a broader mechanism governing yolk component incorporation.

Despite the pronounced effect on yolk uptake, we did not observe changes in oviposition rates following *VgR* silencing. A similar phenotype was previously reported for *Vg*-silenced females, in which females laid normal numbers of eggs that were nevertheless yolk-depleted [61]. Thus, direct depletion of Vg or its receptor does not disrupt the signals that instruct the ovary to produce eggs. This contrasts with the knockdown of the JH receptor Methoprene-tolerant (Met) in *R. prolixus*, where silencing markedly reduces egg production [86]. These findings support a hierarchical organization of reproductive control, in which endocrine regulators such as JH are able to coordinate oocyte development, encompassing oocyte differentiation, meiotic progression, oocyte maturation/activation, etc. while operating independently of yolk load. This refines our understanding of how oogenesis is regulated in triatomines. Furthermore, to determine whether the reproductive phenotypes observed upon *VgR* silencing could be secondary to systemic endocrine imbalances, we measured representative effectors of the JH and ecdysone signaling pathways, which are known regulators of vitellogenesis [36–40]. Because dsRNA treatment is systemic, potential endocrine perturbations could indirectly affect ovarian physiology. Overall, we did not detect consistent reductions in these transcription factors. Although some statistically significant differences were observed, their magnitude was small and, when present, tended toward mild upregulation of certain ecdysone-responsive genes. These modest changes suggest that major endocrine disturbances are unlikely to account for the ovarian phenotypes and are consistent with the observation that oviposition rates were not altered.

Although *VgR* silencing caused a marked depletion of yolk proteins, the other major energy reserves for the embryo, glycogen and TAG, remained unchanged, indicating that their accumulation occurs independently of the VgR pathway. Yet, these reserves alone are apparently insufficient to sustain embryonic development. Notably, phospholipid and cholesterol levels, essential for membrane formation, were reduced in eggs from *VgR*-silenced females, likely because of the impaired buildup of internal membranes to sustain the YG biogenesis. Plus, DAG and phospholipids are known constituents of Vg [5], so the lack of Vg accumulation might also account for some of the observed reduction of those lipids.

The loss of VgR also led to alterations in the microbial profile of the oocytes. The reduction in bacterial 16S rRNA levels and the concomitant expansion of several members of the core virome highlight a potential link between yolk endocytosis and the vertical transmission of microbial components. In several insects, both bacteria and viruses have been shown to exploit reproductive pathways to reach developing oocytes. For instance, in *Drosophila melanogaster*, the endogenous retrovirus *Zam* is translocated from the follicular epithelium to the oocyte via vitellogenin trafficking [87, 88]. Similarly, in the green rice leafhopper *Nephotettix cincticeps*, the rice dwarf virus attaches to the endosymbiont *Sulcia* to facilitate oocyte entry [89], while in *L. striatellus*, the rice stripe virus hijacks the VgR pathway to achieve vertical transmission [27, 28]. In whiteflies, the tomato yellow leaf curl virus binds directly to vitellogenin to access the ovaries [90, 91]. Vitellogenin itself is known to bind bacteria via pathogen-associated molecular patterns (PAMPs), functioning as a carrier molecule that can mediate bacterial transport into the oocyte [59, 92].

Interestingly, we found that *VgR* silencing favored viral expansion while decreasing bacterial abundance in the oocytes, a pattern not recapitulated by *Vg* silencing. Instead, *Vg*-silenced oocytes exhibited reduced viral loads and only a mild decrease in bacterial abundance. Because both knockdowns generate yolk-depleted eggs, the distinct microbial patterns cannot be explained by the absence of yolk proteins in the oocytes per se. Differences in circulating Vg concentrations between the two conditions also failed to account for the observed phenotypes, as viral and bacterial loads in the hemolymph were comparable. Furthermore, purified *R. prolixus* Vg showed no direct antimicrobial or antiviral activity against *E. coli*, *S. aureus*, or Mayaro virus in vitro. In addition, neither immune gene modulation (defensins) nor RNAi pathway activation appears to be the primary cause of the microbial changes, as both correlated negatively with bacterial and viral loads. This inverse relationship suggests that the observed transcriptional changes are more likely a consequence of the altered microbial levels rather than their cause. Altogether, these observations indicate that the altered microbial balance arises from the absence of functional VgR itself, rather than from yolk depletion or modulation of canonical immune pathways.

Assuming that the main determinants of the microbial alterations observed are VgR-related, it remains challenging to discern whether these changes arise primarily from altered entry (flux) of the microbes into the oocytes, or whether the differential flux of other components secondarily results in altered microbial expansion within the oocyte. Nevertheless, some mechanistic possibilities can be proposed. One possibility is that VgR itself acts as an entry or regulatory platform for additional yet unidentified ligands that influence microbial flux and/or the maintenance within the oocyte. In this scenario, the absence of VgR would prevent the entry of one or more factors that normally disfavor viral expansion while sustaining bacterial presence, thereby shifting the microbial balance toward reduced bacterial load and enhanced viral abundance. Another possibility is that the reduction in VgR likely causes a severe alteration of the endocytic environment of the oocyte cortex, since the loss of endocytic activity required for the massive YG biogenesis probably disrupts the balance between membrane influx and recycling of surface proteins. Consequently, the repertoire of surface proteins available to mediate uptake events would change, potentially resulting in distinct fluxes of ligands or microbial particles into the oocyte through alternative, non-VgR-mediated routes.

Plus, when comparing *VgR*- and *Vg*-silencing data, the opposite viral trends observed in *Vg*-silenced oocytes when compared to *VgR*-silenced oocytes may also reflect differences in VgR localization and/or availability at the oocyte membrane. Under *Vg* silencing, the lack of VgR’s major ligand (Vg) likely increases the number of unoccupied VgR molecules at the oocyte surface, which could enhance receptor activity and facilitate the internalization of other VgR-interacting molecules, thus resulting in the opposite effect of silencing VgR. Moreover, the reduction in endocytic flux may further increase the residence time of VgR at the membrane. In this context, the higher abundance of unoccupied VgR molecules in the oocyte surface could underlie the opposing viral outcomes between *VgR* and *Vg* knockdowns, suggesting that the changes observed upon Vg silencing might stem from increased VgR availability to alternative ligands.

Altogether, these observations suggest that VgR functions as a central node connecting endocytic dynamics with microbial accumulation in the developing oocyte, acting not only as a cargo carrier but also as a signaling receptor that coordinates these processes. However, a limitation of this study is that gene expression analyses are based on transcript levels without protein validation, and thus should be interpreted with caution. Although the hypotheses proposed above are intriguing, they remain speculative at this stage. It is technically challenging to design experimental approaches capable of disentangling whether the observed microbial changes arise from altered entry, expansion, or survival within the oocyte. Future studies specifically addressing these mechanisms will therefore be necessary to establish causality. It would also be particularly interesting to explore how such alterations may influence vertical transmission and progeny viability. Brito et al. (2021) observed that all RpVs identified were markedly reduced during *R. prolixus* embryogenesis and proposed that this decline results from an upregulated RNAi response aimed at lowering viral levels to support normal embryo development, while still maintaining residual viral populations that later re-expand during the early nymphal stages. If the balance of viral load is indeed finely tuned to sustain potential symbiotic benefits, then the crosstalk between yolk accumulation and microbial inheritance may represent a key mechanism not only for ensuring progeny fitness but also for shaping broader aspects of species ecology, including habitat adaptation, interspecific interactions, and long-term vector-microbe coevolution.

## Conclusions

Together, these findings indicate that VgR function extends beyond yolk accumulation to encompass a broader role in the molecular crosstalk between reproduction and microbial inheritance. This expanded functional framework highlights an unrecognized layer of complexity in the reproductive biology of triatomines.

## Methods

### Insects and eggs

Adult insects and eggs were kept in a controlled insectarium, with a 12-h light/dark photoperiod, temperature of approximately 28 °C, and relative humidity around 70–80%. As adults, the insects were fed every 21 days with rabbit blood. All procedures follow a protocol approved by the Ethics Committee on the Use of Animals (CEUA-UFRJ), registered under CEUA #123/22.

### Bioinformatics

The *VgR* sequence of *R. prolixus* was obtained through the transcriptome of several tissues involved in egg production [23] and validated using the genome and transcriptome deposited in the VectorBase database (Rpro C3.2) (www.vectorbase.org). Conserved domains were predicted using the online software SMART (https://smart.embl-heidelberg.de/).

### Dissection of the ovary parts, follicles, and chorionated oocytes

The different parts of the ovariole were carefully dissected in phosphate buffered saline (PBS) 137 mM NaCl, 2.7 mM KCl, 10 mM Na_2_HPO_4_, and 1.8 mM KH_2_PO_4_, pH 7.4 using fine tweezers and dissecting scissors under the stereo microscope according to [93], and the structures (tropharium, previtellogenic and vitellogenic follicles, and chorionated oocytes) were classified by length and morphology according to [94, 95]. For the previtellogenic follicles, it is not possible to rule out contamination from the tropharium or adjacent oocytes, thus these samples must be interpreted as fractions enriched in previtellogenic oocytes. All ovary parts were dissected 7 days after blood meal.

### Total RNA extraction and cDNA synthesis

For total RNA extraction, the midgut (MG), fat body (FB), and reproductive organs (ovary or testis (OV or TEST)) were dissected 7 days after blood feeding from adult females and males. All samples were homogenized with the assistance of a plastic potter in Trizol reagent, and the RNA was extracted, measured by spectrophotometry in NanoDrop (Thermo Scientific) at 260 nm and treated with DNase I (Invitrogen). Following, the treated RNAs were used in reverse transcription reactions using the High-Capacity cDNA Reverse Transcription Kit (Applied Biosystems), according to the manufacturer’s protocol.

### PCR/qPCR

PCR reactions used *R. prolixus VgR*-specific primers previously described (Faria-Reis et al., 2023) to amplify 216 base pair fragments using the following cycling: 10 min at 95 °C, followed by 35 cycles of 30 s at 95 °C, 30 s at 52 °C and 1 min at 72 °C and a final extension of 15 min at 72 °C. qPCR reactions were carried out in a StepOne Real-Time PCR System thermocycler (Applied Biosystems), using SYBR Green PCR Master Kit (Applied Biosystems) and the following parameters: 95 °C for 10 min, 40 cycles at 95 °C for 15 s and 60 °C for 1 min. The relative expressions were calculated using the delta Ct (cycle threshold) obtained using the endogenous genes 18 s (RPRC017412) or EF1 (RPRC015041) and expressed as 2^−dCt^ or 2^−ddCt^, depending on the experiment [96]. Under our experimental conditions, both endogenous genes showed stable expression, which supported their use as reference genes in accordance with the MIQE guidelines [97]. The sequences of all primers are described in Additional File 1: Table S1.

### Gene silencing via RNAi

Double-stranded RNAs (dsRNAs) were synthesized by the MEGAscript RNAi Kit (Ambion Inc.), using primers designed for specific sequence amplification with the T7 promoter. The dsRNA was designed to generate amplicons of 707 base pairs. The *Escherichia coli* MalE gene encodes a maltose-binding protein and was used as a control dsRNA. Using a Hamilton syringe, each insect was injected with 1 µg of control or experimental dsRNA, according to the following protocols: (1) for mating experiments between *VgR*-silenced males and wild-type virgin females, dsRNA was injected in the males 13 days before blood feeding. Eight days later, silenced males and virgin females are allowed to mate for 5 days before being blood fed; (2) for all other experiments, adult insects were injected 2 days before blood feeding. Silencing efficiency was subsequently confirmed by qPCR 7 days after dsRNA injection (protocol 1) and 7 days after feeding (protocol 2). The sequences of the primers are described in Additional File 1: Table S1.

### Gene silencing phenotype evaluation

After dsRNA injection according to protocol 2, fed males and females were transferred to individual vials. For the digestion experiments, the insects were weighed immediately before blood feeding (day 0) and subsequently at defined time points after feeding. The first post-feeding measurement was performed on day 3. Thereafter, body weight progressively declined due to ongoing blood digestion and excretion, as insects were maintained without additional feeding until day 21. For oviposition analysis, eggs laid by individual insects were collected on the same days that the animals were weighed, and approximately 2 weeks later, the nymphs that hatched were counted. Mortality rates were recorded daily.

### Fertilization status

To determine if fertilization was occurring in control and silenced eggs, a PCR was performed targeting a male-specific DNA sequence (chromosome Y) (GenBank: JX559072.1), as previously reported [49]. Briefly, freshly laid eggs from control and silenced individuals were collected, and genomic DNA was phenol extracted and precipitated with 100% ethanol and 2 M ammonium acetate. Purified genomic DNA samples were used as templates for amplification using specific primers. The fat body of adult male insects was used as a positive control, and eggs laid by non-mated females were used as a negative control. The PCR product was subsequently visualized in a 2% agarose gel.

### Hemolymph extraction and SDS-PAGE

Following dsRNA injections, hemolymphs from females were collected on day 7 after blood feeding. Once extracted, the hemolymph was diluted 2 × in 50 mM HEPES buffer, pH 7.4 containing a cocktail of protease inhibitors (aprotinin 0.3 µM, leupeptin 1 µg/µl, pepstatin 1 µg/µl, PMSF 100 µM and EDTA 1 mM) and approximately 8 mg of phenylthiourea. 0.5 µl of each hemolymph sample was loaded in a 10% and 13.5% SDS-PAGE and stained with Coomassie Blue or silver nitrate [98]. Each biological replicate corresponds to the hemolymph of one insect.

### Egg homogenates and SDS-PAGE

Eggs were collected 0–24 h after oviposition from females injected with dsMal and dsVgR and homogenized in 50 μl of 50 mM pH 7.4 HEPES buffer containing protease inhibitors (aprotinin 0.3 μM, leupeptin 1 μg/μl, pepstatin 1 μg/μl, PMSF 100 μM, and EDTA 1 mm). The equivalent of one-tenth of the egg was loaded in a 10% and 15% SDS-PAGE and stained with silver nitrate. For each biological replicate, samples were prepared using a pool of 3 eggs, each laid by a different female.

### Determination of protein content

Hemolymph and egg samples were collected as described above and the total amount of protein levels was measured by the Lowry method (Folin), using 1–7 µg of BSA as a standard [99] in an E-MAX Plus microplate reader (Molecular devices) using SoftMax Pro 7.0.

### Hemolymph RHBP titration

RHBP quantification in the hemolymph was performed as described by Walter-Nuno et al. [49]. Briefly, hemolymph samples (5 µL) were collected from females and immediately diluted in 500 µL PBS containing 3–13 µg/mL phenylthiourea to prevent melanization. The absorbance of the Soret band was monitored between 350 and 450 nm, and the value at hemin saturation, determined from a break in the titration curve, was used to calculate the total RHBP concentration based on the molar extinction coefficient of 0.0645 µM.

### Vitellogenin (Vg) purification

Vg was purified from *R. prolixus* oocytes following the ammonium sulfate fractionation and gel-filtration procedure described by [42], with minor modifications. Briefly, oocytes collected 4 to 6 days after a blood meal were homogenized in 20 mM Tris–HCl (pH 7.0) containing 0.15 M NaCl and a cocktail of protease inhibitors. The homogenate was centrifuged at 11,000 × g for 5 min at 4 °C, and the lipid layer and pellet were discarded. Solid ammonium sulfate was added to the supernatant to reach 45% saturation, and the suspension was gently stirred for 20 min at 4 °C. After centrifugation (11,000 × g, 10 min), the pellet was discarded, and the supernatant was brought to 60% saturation. The resulting precipitate was washed twice with 60% ammonium sulfate and re-extracted by resuspending in a 45% saturated solution followed by centrifugation. The supernatant was dialyzed against 0.15 M NaCl, 10 mM Tris–HCl (pH 7.0), and applied to a Sephadex G-200 column (2.5 × 55 cm) equilibrated with the same buffer. Protein content of each fraction was monitored at 280 nm, and the characteristic Vg fractions were pooled and dialyzed against deionized water.

### Bacterial growth inhibition assay

Vg antibacterial activity was tested following the protocol of [100] with minor modifications. The antibacterial activity of purified *R. prolixus* Vg was evaluated against *E. coli* ATCC 25922 and *S. aureus* ATCC 29213. Bacteria were cultured overnight in Luria–Bertani (LB) medium at 37 °C with shaking, then suspended in sterile saline (0,85%) to reach 0.5 MacFarland, diluted at a 1:200 rate in cation-adjusted Mueller–Hinton (CAMH) broth, and distributed into sterile 96-well plates. Purified Vg was serially diluted in CAMH and added to the bacterial suspensions to a final concentration of 12.5–200 µg/mL. Bacterial growth was quantified after 24 h at 37 °C with shaking by measuring OD₆₀₀ using a microplate reader.

### Trypanosoma rangeli infection

*T. rangeli* infection assays were conducted following the procedures previously described [72]. Briefly, adult females of *R. prolixus* were infected by injection into the hemocoel of 2 µL of epimastigotes of *T. rangeli* (1 × 10^4^ parasites/mL) in sterile saline, 3 days after the blood feeding. On the third day after infection, the insects were dissected, and the fat body and ovary were used for the RT-qPCRs.

### Vg antiviral activity—virus propagation and Mayaro plaque assay

The arbovirus MAYV (MAYV BR/SJRP/LPV01/2015 strain) was propagated in *Aedes albopictus* C6/36 cells. The culture supernatant was collected, centrifuged, aliquoted, and stored at − 80 °C for subsequent use in cell infection experiments. Viral titers were determined by plaque assay using Vero cells (African green monkey kidney). Cells were maintained in DMEM (ThermoFisher) supplemented with 10% FBS, 7.5% sodium bicarbonate, and 1% L-glutamine. They were seeded as monolayers (~ 70% confluence) in 24-well plates and incubated at 37 °C with 5% CO₂ for approximately 24 h before the assay. Aliquots of MAYV (100 µL, 5 × 10⁸ PFU/ml) containing either Tris/HCl 10 mM pH 7.4 (control) or Vg (400 µg/mL) were added to the respective cell monolayers. Subsequently, 500 µL of DMEM containing 2% FBS and 1% methylcellulose (Sigma-Aldrich) were added to each well. Plates were incubated at 37 °C with 5% CO₂ for 44 h. Cells were then fixed with a 10% formaldehyde solution (Sigma-Aldrich) for 1 h. Then, they were washed and stained with crystal violet (0.5%) in a 20% methanol solution for 20 min at room temperature. Excess stain was removed by washing with water.

### Lipid extraction and thin-layer chromatography (TLC)

Zero to 24 h control and silenced eggs were used for lipid extraction. The lipid composition was analyzed by thin-layer chromatography (TLC) on silica gel plates (Merck) using two consecutive solvent systems [101]. Plates were stained with copper reagent, and relative lipid composition was determined by densitometry using TotalLab Quant v11 (TotalLab) with background corrections after comparison with commercial lipid standards (Sigma) [102]. A total of 5 eggs were used for each biological replicate.

### Determination of glycogen content

Egg homogenates were prepared with 5 eggs in 100 μL of lysis buffer containing 200 mM sodium acetate buffer, pH 4.8, and 0.001% Triton X-100 supplied with a protease inhibitors cocktail (Sigma #P8340) and were used to determine glycogen content. For 4 h, the experimental samples were incubated at 40 °C in the presence of 20 μL (1U) of amyloglucosidase, except for the controls, which were prepared under the same conditions but without enzyme. The quantification was made using the Glucox 500 kit (Doles reagents), according to the manufacturer’s instructions.

### Light microscopy

Eggs collected 0–24 h after oviposition were gently disrupted using fine tweezers in 2 μL of PBS on a slide to observe the yolk granules (YGs) suspension as previously reported [103, 104]. The samples were observed using a Zeiss Axio Imager D2 microscope equipped with a Zeiss Axio Cam MRc 5 digital camera operated in differential interferential contrast (DIC) mode.

### Yolk granules flow cytometry

Suspensions of YGs were obtained by gently disrupting freshly dissected chorionated oocytes in PBS (2 oocytes in 250 μL of buffer) using a plastic pestle. Population profiles of YGs were acquired in a FACS Calibur equipment (BD Bioscience) powered by CellQuest Pro v5.1 software and analyzed using Flowing Software 2.5.1. For each biological replicate, samples were prepared using a pool of 2 eggs from different insects.

### Statistics

Student’s *t*-test was used for the comparison of two different conditions, and one-way ANOVA or two-way ANOVA followed by Tukey’s multiple comparisons for the comparison among more than two conditions. Log-rank (Mantel-Cox) test was performed for the survival experiments. All the tests used the GraphPad Prism 8 software. Differences were considered significant at *p* < 0.05.

## Supplementary Information

Additional file 1: Figure S1: Original SDS-PAGE of hemolymph samples. Figure S2. Densitometric analysis of hemolymph proteins. Figure S3. Original SDS-PAGE of egg/embryo samples. Figure S4. Original thin layer chromatography (TLC) plates of lipid extracts. Figure S5: Male *VgR* expression is dispensable for fertility and progeny viability. Figure S6: Purified Vg does not affect Mayaro virus infectivity in cell culture. Table S1: Genes and primers list. Table S2. Relative growth of *Escherichia coli* in the presence of different concentrations of Vg Table S3. Relative growth of *Staphylococcus aureus *in the presence of different concentrations of Vg.

## Data Availability

All data generated or analyzed during this study are included in this published article and its supplementary information files.

## Abbreviations

AMP: Antimicrobial peptide
Ary: Arylphorin
BR-C: Broad-complex
CAMH: Cation-adjusted Mueller–Hinton broth
CHO: Cholesterol
DAG: Diacylglycerol
DIC: Differential interference contrast
DMEM: Dulbecco’s modified Eagle medium
dsMal: Double-stranded RNA targeting *Escherichia coli* MalE gene (control)
dsRNA: Double-stranded RNA
dsVg: Double-stranded RNA targeting vitellogenin
dsVgR: Double-stranded RNA targeting vitellogenin receptor
ECDF: Ecdysone early response factor
EGF: Epidermal growth factor-like domain
EGF_CA: Calcium-binding EGF-like domain
EGFD: Epidermal growth factor-like domain
EF1: Elongation factor 1
FB: Fat body
FBS: Fetal bovine serum
FSC: Forward scatter
HC: Hydrocarbon
HR-3: Hormone receptor 3
HR-4: Hormone receptor 4
InsR: Insulin receptor
JH: Juvenile hormone
Kr-h1: Krüppel homolog 1
LB: Luria–Bertani medium
LDLa: Low-density lipoprotein receptor class A domain
LDLR: Low-density lipoprotein receptor
LBD: Ligand-binding domain
Lp: Lipophorin
MAG: Monoacylglycerol
MAYV: Mayaro virus
Met: Methoprene-tolerant
MG: Midgut
NTD: Neglected tropical disease
OV: Ovary
PBS: Phosphate-buffered saline
PFU: Plaque-forming units
PL: Phospholipid
qPCR: Quantitative polymerase chain reaction
RHBP: Rhodnius heme-binding protein
RISC: RNA-induced silencing complex
RNAi: RNA interference
RpV: *Rhodnius prolixus* Virus
RSV: Rice stripe virus
RT-qPCR: Reverse transcription quantitative PCR
SSC: Side scatter
TAG: Triacylglycerol
TLC: Thin-layer chromatography
Vg: Vitellogenin
Vg1: Vitellogenin isoform 1
Vg2: Vitellogenin isoform 2
VgR: Vitellogenin receptor
Vit: Vitellogenic
Vt: Vitellin
vsiRNA: Viral small interfering RNA
YG: Yolk granule
