# Supplementary material for: Loss of vitellogenin receptor function results in yolk depletion, virome expansion, and reduced bacterial load within the oocytes of Rhodnius prolixus
Source: BMC Biol. 2026 May 13;24:151. doi: 10.1186/s12915-026-02622-7 (PMC13340397; doi:10.1186/s12915-026-02622-7)
Supplement: Supplementary file 1 — Additional file 1: Figure S1: Original SDS-PAGE of hemolymph samples. Figure S2. Densitometric analysis of hemolymph proteins. Figure S3. Original SDS-PAGE of egg/embryo samples. Figure S4. Original thin layer chromatography (TLC) plates of lipid extracts. Figure S5: Male VgR expression is dispensable for fertility and progeny viability. Figure S6: Purified Vg does not affect Mayaro virus infectivity in cell culture. Table S1: Genes and primers list. Table S2. Relative growth of Escherichia coli in the presence of different concentrations of Vg Table S3. Relative growth of Staphylococcus aureus in the presence of different concentrations of Vg. [file 12915_2026_2622_MOESM1_ESM.docx]

## Supplementary Data:


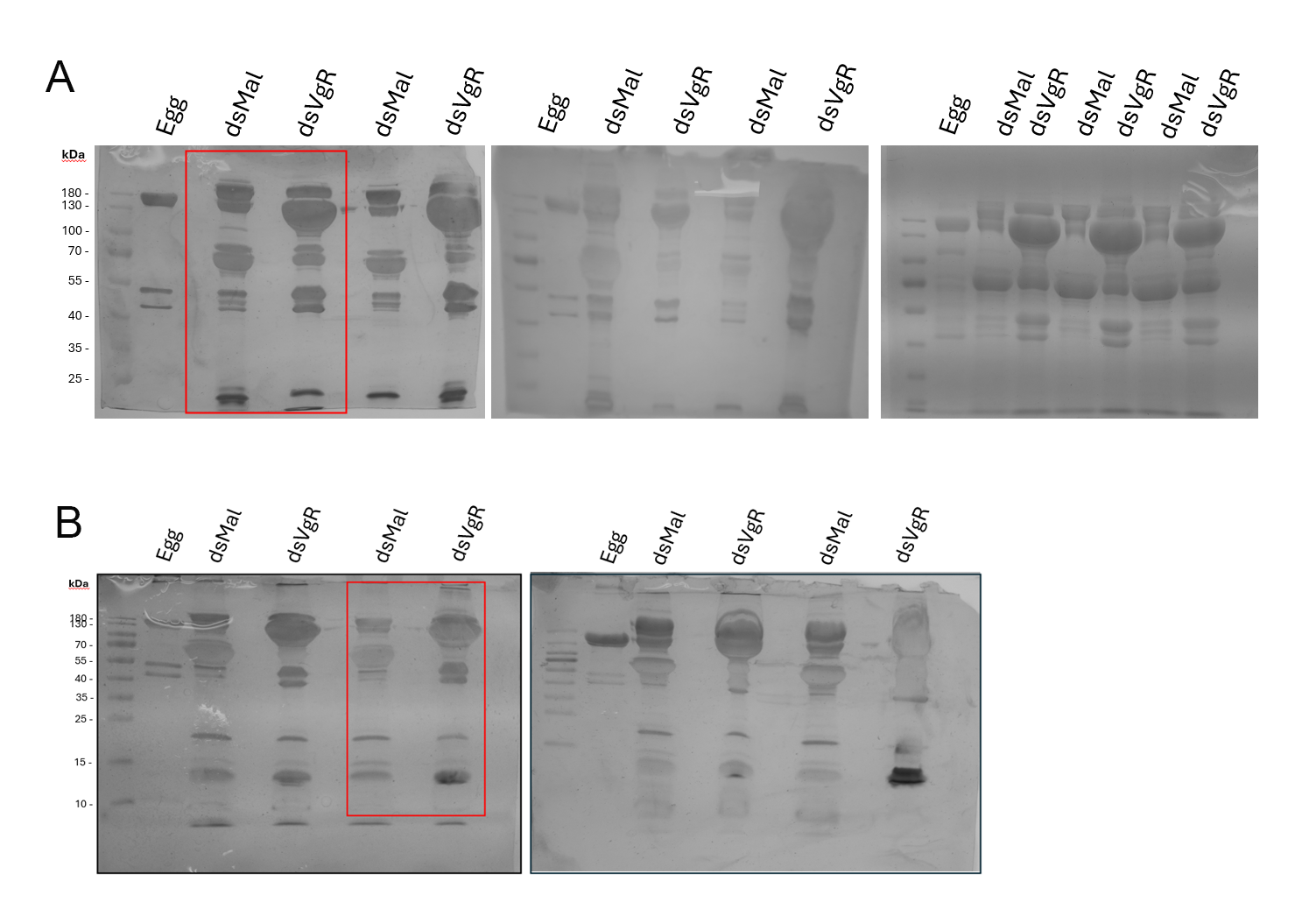


**Figure S1. Original SDS-PAGE of hemolymph samples.** SDS-PAGE profiles of hemolymph proteins showing the original, uncropped gels used for Figure 3B. **(A)** 10% gels; **(B)** 13,5% gels. The red boxes indicate the lanes that were cropped and presented in the main figure.


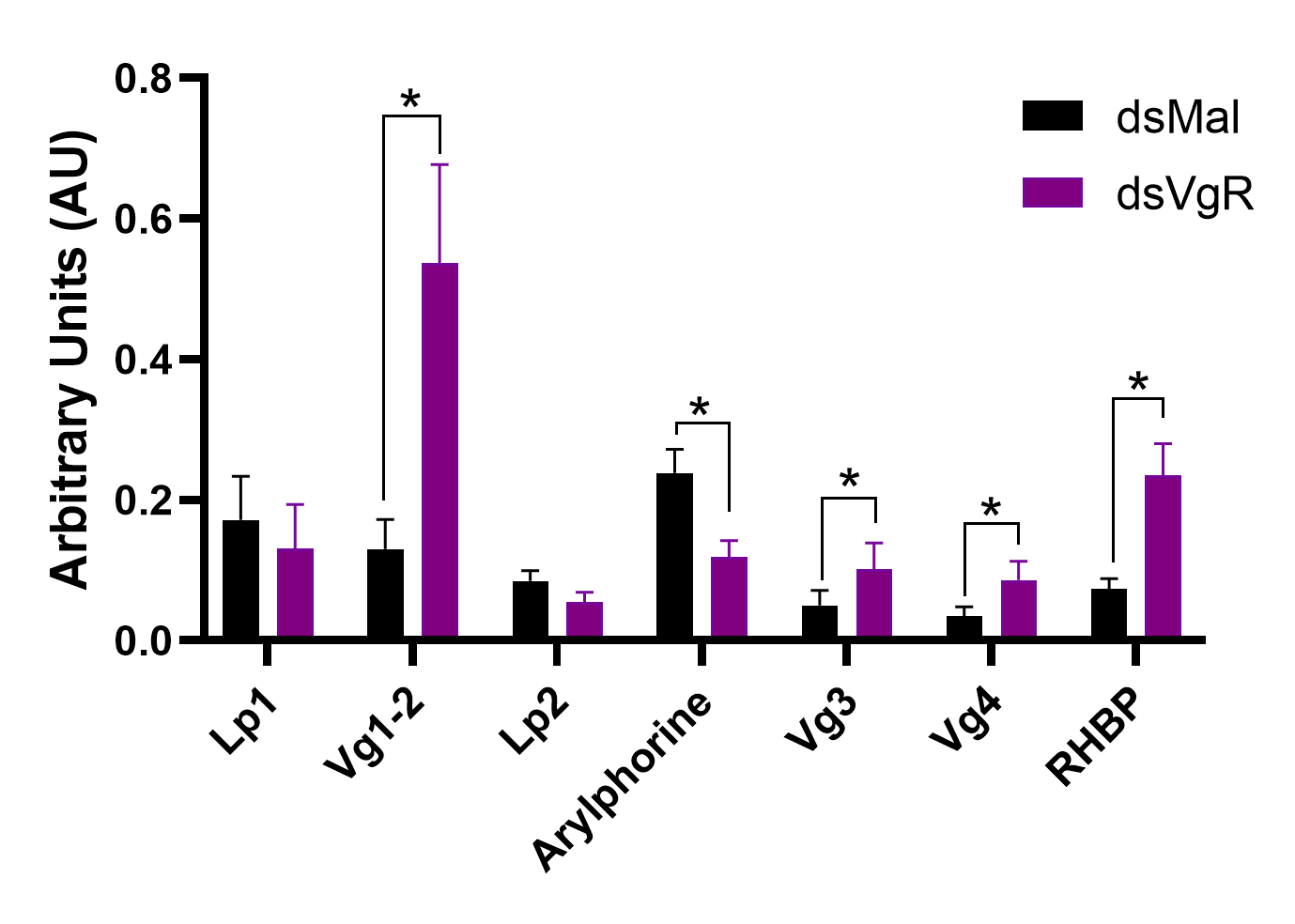


**Figure S2. Densitometric analysis of hemolymph proteins.** Quantification of band intensities corresponding to vitellogenin (Vg), Rhodnius heme binding protein (RHBP), lipophorin (Lp), and arylphorin (Ary) from SDS-PAGE gels shown in Figure 3B. Lp1, Lp apoprotein 1; Lp2, Lp apoprotein 2. Vg1-2, Vg apoprotein 1 and 2; Vg3, Vg apoprotein 3; Vg4, Vg apoprotein 4. Vg and RHBP levels were significantly increased in 2 to 3-fold in VgR-silenced insects compared to controls. Ary levels were significantly reduced by approximately 40%, whereas Lp levels did not show significant changes. T-test. All graphs show mean ± SEM.*p<0.05.


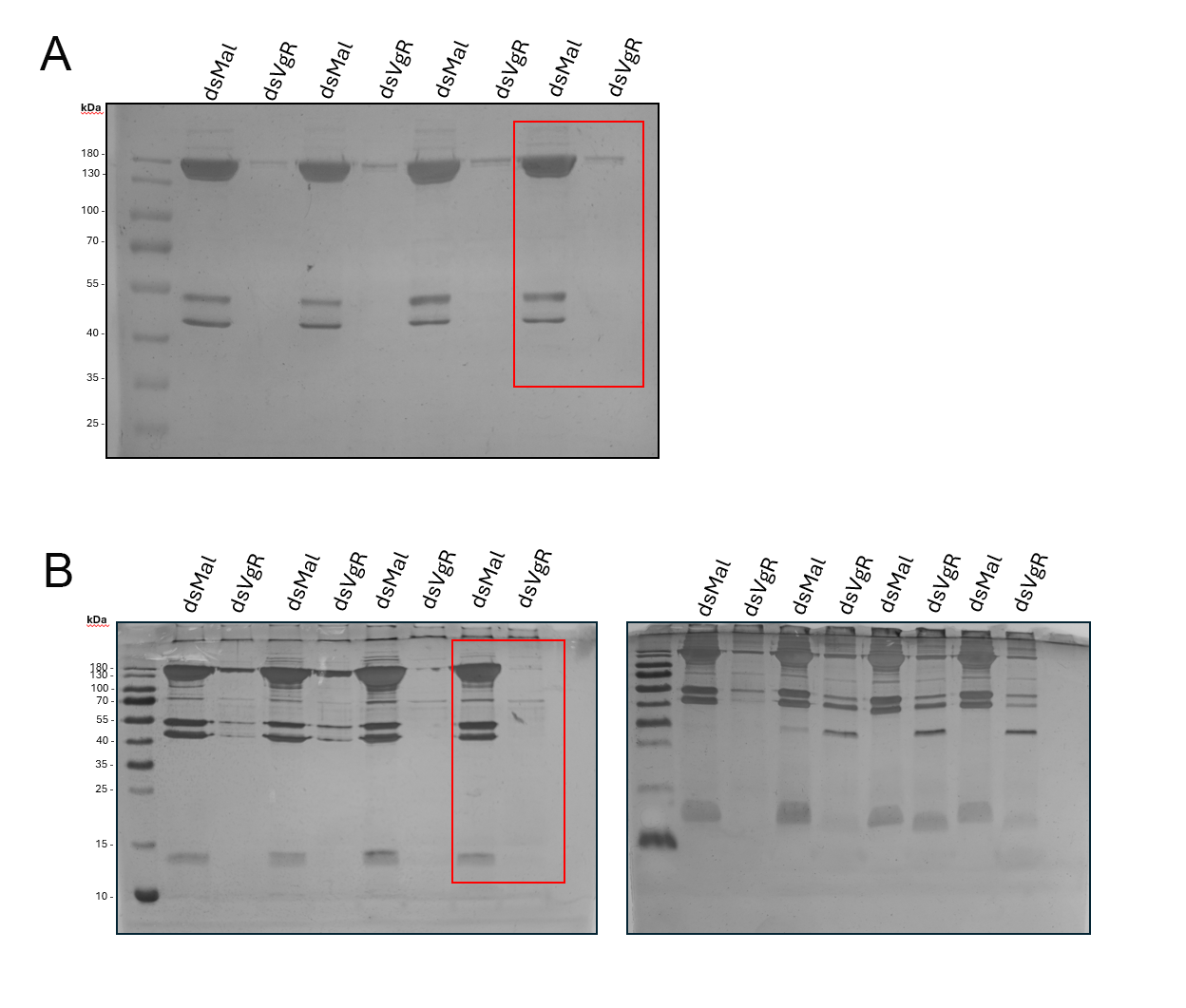
**Figure S3. Original SDS-PAGE of egg/embryo samples.** SDS-PAGE profiles of proteins from eggs/embryos showing the original, uncropped gels used in figure 4G. (A) 10% gel; (B) 15% gel. Red boxes indicate the lanes that were cropped and presented in the main figures.


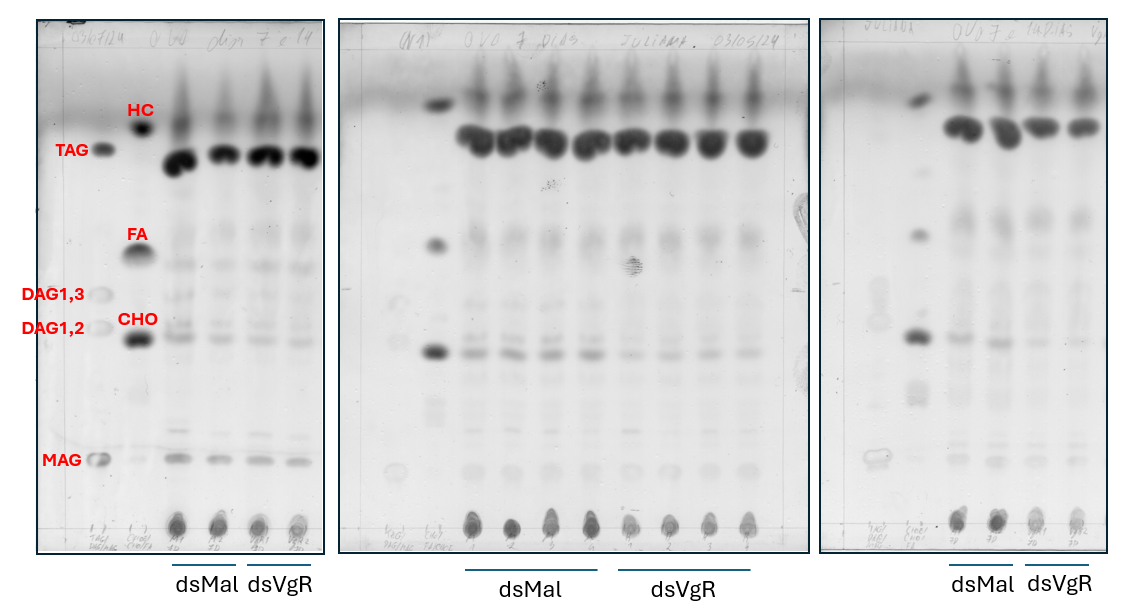


**Figure S4. Original TLC plates of lipid extracts.** Representative thin-layer chromatography (TLC) profiles showing the original, uncropped plates used for lipid analysis in Figure 4I. Lipid classes were identified based on migration relative to standards.

**
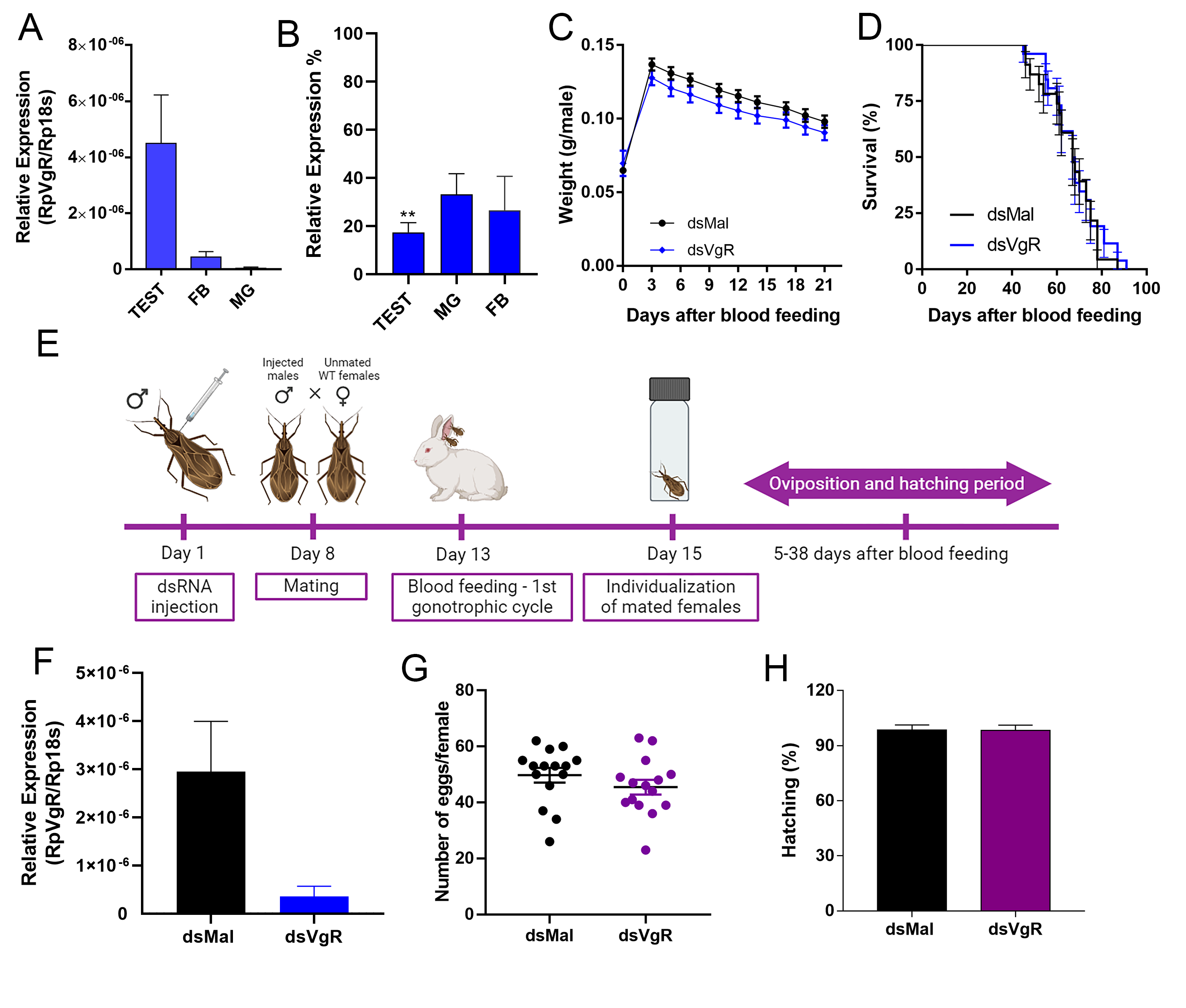
**

**Figure S5**: **Male *VgR* expression is dispensable for fertility and progeny viability. (A)** RT-qPCR showing the expression profile of *VgR* in the different tissues of adult males dissected 7 days after blood feeding (n=5-8). One-way ANOVA. **(B)** RT-qPCR shows the silencing efficiency for *VgR* in different organs after injection of dsRNA designed to target *VgR* (n=3-7). T-test. Fat body (FB), midgut (MG), and testis (TEST). **(C)** Effects of *VgR* silencing on male digestion/diuresis, showing their weight during the gonotrophic cycle (n=30). Two-Way ANOVA. p>0.05. **(D)** Survival rates of control and Vg-silenced females (n=30). Log-rank (Mantel-COX) test. p>0.05. **(E)** Experimental design for dsRNA injections in the mating experiments. Adult males were injected with VgR dsRNA 13 days before blood feeding. On day 8 after dsRNA injection, the silenced males were allowed to mate with virgin females for 5 days. After the mating period, both males and females were blood-fed. **(F)** Silencing efficiency for the males on day 7 after dsRNA injection (before mating) is presented in the testis (n=5). T-test. P=0.067. **(G)** Total oviposition of females mated with control and *VgR* silenced males (n=15). T-test p>0.05. **(H)** Hatching rates of eggs laid by females mated with control and *VgR* silenced males (n=15). T-test. p>0.05. All graphs show mean ± SEM.*p<0.05, **p<0.01.

##
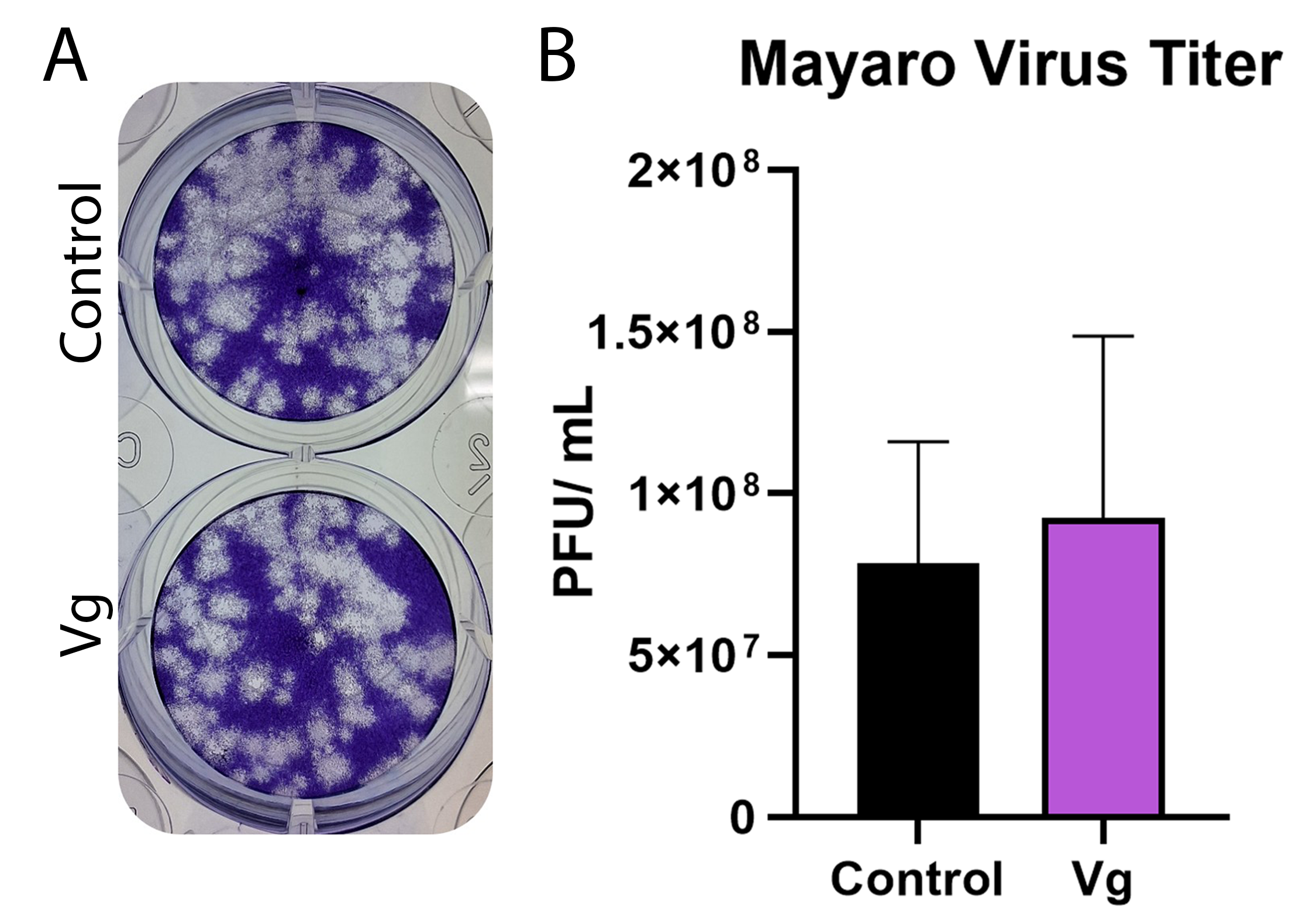


**Figure S6: Purified Vg does not affect Mayaro virus infectivity in cell culture.
(A)** Representative plaque assay showing *Mayaro virus* (MAYV) infection in Vero cell monolayers incubated with vehicle (Control) or purified Vg (400 µg/ml). Viral plaques were visualized by crystal violet staining. **(B)** Quantification of viral titers expressed as plaque-forming units per milliliter (PFU/mL). Bars represent mean ± SEM from three independent experiments (n=3). t-test; *p* > 0.05.

## Table S1: Genes and primers list. All sequences were obtained from *Vector base* (<https://www.vectorbase.org/>) or previous works and primers were synthesized by Macrogen or IDT technologies.

| **Gene** | **Vector Base** | **Primer Sequence (5’ – 3’)** | **Reference** |
| --- | --- | --- | --- |
| **18S (qPCR)** | RPRC017412 | FOR: TCGGCCAACAAAAGTACACA  REV: TGTCGGTGTAACTGGCATGT | **Majerowicz et al.,** 2011 |
| **VgR (qPCR)** | RPRC000551 | FOR: TTACAGCATATCGTCCTCCG  REV: GGGAGAACGGCAGACATTGT | Faria-Reis et al., 2023 |
| **Kr-H1 (qPCR)** | RPRC014398 | FOR: ACAACCTGTAGTGGCTGTCG  REV: ACGACACGCTACAGTGTACG | Leyria et al., 2022 |
| **E75 (qPCR)** | RPRC000853 | FOR: AGGTTTTCACCAGCCATCAG  REV: GCCCGCTTTCAAAAGTGTAA | Benrabaa et al., 2023 |
| **E74 (qPCR)** | RPRC007565 | FOR: ACCGGGAGAAGGGTGTATTT  REV: AGAGCACGTCCCATTGTTTC | Benrabaa et al., 2023 |
| **BR-C (qPCR)** | RPRC003967 | FOR: TCGAGGACCTACATGCTCTTG  REV: TTGTCCAAACCCATGGAGAC | Benrabaa et al., 2023 |
| **HR-3 (qPCR)** | RPRC003681 | FOR: GTGTGCGGAGACAAATCAAG  REV: CATGCCCAGCCTTAGACATT | Benrabaa et al., 2023 |
| **HR-4 (qPCR)** | RPRC012796 | FOR: TGAAAGGACTAGCGGGAGAA  REV: CATTGCTAACGTGGCGTCTA | Benrabaa et al., 2023 |
| **FTZ-F1 (qPCR)** | RPRC002968 | FOR: GGATTAGCATCACCTGGCATA  REV: GCTTTCGCAAGAGAAGATGC | Benrabaa et al., 2023 |
| **RpV1 (qPCR)** | GenBank: MZ328304 | RpV1_1p: GACACAAGCTTGTGATACAATC  RpV1_1m: ATOTGGTAGACCTATAGATTC | De Brito et al., 2021 |
| **RpV4 (qPCR)** | GenBank: MZ328307 | RpV4_1p: TCCTGTTCATCTGCTTCTGC  RpV4_1m: CAACTACGCTTGTTCCATGTG | De Brito et al., 2021 |
| **RpV5 (qPCR)** | GenBank: MZ328308 | RpV5_1p: GTTCAAGCGTAGAACTATGTC  RpV5_1m: CACGAGCAAACGTGTTTGAC | De Brito et al., 2021 |
| **RpV6 (qPCR)** | GenBank: MZ328309 | RpV6_1p: ATCATTTCCGTGTCCAGCAG  RpV6_1m: ATCATAGCAGCATAGTGCATC | De Brito et al., 2021 |
| **RpV7 (qPCR)** | GenBank: MZ328310 | RpV7_1p: GATGATTACCGGCGCATTTTC  RpV7_1m: ATCCGTCTCCACGTGCATC | De Brito et al., 2021 |
| **16S rRNA (qPCR)** | GenBank: KT726373 | FOR: ′TTCGGTGGGAAAGAAAGTTTC3  REV: TAGGGCTTTCACATTCGACTTA-AAT | Guizzo et al., 2017 |
| **EF1 (qPCR)** | RPRC015041 | FOR: GATTCCACTGAACCGCCTTA  REV: GCCGGGTTATATCCGATTTT | **Majerowicz et al.,** 2011 |
| **Def A (qPCR)** | RPRC012185 | FOR: GAATACTCCACTCAACCGCAAC  REV: agggcatcatctagttgttgatgagtg | Vieira et al. 2016 |
| **Def B (qPCR)** | RPRC004803 | FOR: GGATATTCCACTCAACCGCAAC  REV: agagcatcgtctaattcttgttgagtg | Vieira et al. 2016 |
| **Def C (qPCR)** | RPRC012184 | FOR: CAGTACAGTCCTAATACCTAGCC  REV: tgggcatcatctaattgatgttgagaa | Vieira et al. 2016 |
| **Prol (Prolixicina) (qPCR)** | RPRC013002 | FOR: ACAATTTTGGTGGTGGTTGTC  REV: GCTTGAGCTCTGGTCCTTCC | Ursic-Bedoya et al., 2011 |
| **Dicer 1 (qPCR)** | RPRC002485 | FOR: CTGGCAAATTGTCAAACACC  REV: GAT GAC AAG ATT GAT CCT GC | This work |
| **Dicer 2 (qPCR)** | RPRC013872 | FOR: GCTAGTCTGCTGCCATTG  REV: GTA TGG TGA CAC TCA TCC | This work |
| **Ago 1 (qPCR)** | RPRC013696 | FOR: GGTAACTTAAAGCCAGTATTCG  REV: CCT TCC TTC TAA TGC TTC | This work |
| **Ago 2 (qPCR)** | RPRC002460 | FOR: CTGATGAGGATCGTACAGC  REV: CAT CTG ATA TTC GAG CTG | This work |
| **Ago 3 (qPCR)** | RPRC013054 | FOR: CTCACATAATCGGAGAAACC  REV:  GAC TGT TGT ACG ATC ACC C | This work |
| **Vg 1 (qPCR)** | RPRC013551 | FOR: CGCAGCTACCAAACATGA  REV: TGTGCTTGTTGAGGCTGG | Pereira et al., 2025 |
| **Vg 2 (qPCR)** | RPRC002109 | FOR: CGCTAAGATTTCCCCATG  REV: CCCTGAAACACCTTGGAGCATC | Pereira et al., 2025 |
| **Y chromosome genomic sequence** | ACPB03041887  GenBank: JX559072.1 | FOR: TCCTCCGCCTTGCTTCTCTGT  REV: GTGCGGGCGGTGGATTG | - |
| **VgR (dsRNA)** | RPRC000551 | FOR: ggccgcggGAACGGATGACTTCTCAGAC  REV: cccggggcCAGCCTTACTTCGTCAACAG | This work |
| **Vg1/2 (dsRNA)** | - | FOR: ggccgcggTTCCGTGATGCAGTTGCC  REV: cccggggcGATGAAGTCCAAATTCTTGAGT | Pereira et al., 2025 |
| **T7 adaptor** | - | FOR:GAGAATTCTAATACGACTCACTATAGGGCCGCGG  REV:AGGGATCCTAATACGACTCACTATAGGGCCCGGGGC | - |

## Table S2. Relative growth of *Escherichia coli* in the presence of different concentrations of Vg

| Vg concentration | Relative growth (%) |
| --- | --- |
| 200 µg/mL | 90.18±1.42 |
| 100 µg/mL | 90.20±1.51 |
| 50 µg/mL | 92.49±0.77 |
| 25 µg/mL | 94.90±0.51 |
| 12.5 µg/mL | 99.43±0.06 |

Optical density was measured for *E. coli* cultures incubated with different concentrations of vitellogenin (Vg). Relative growth was calculated in relation to the bacterial control without Vg.

## Table S3. Relative growth of *Staphylococcus aureus* in the presence of different concentrations of Vg

| Vg concentration | Relative growth (%) |
| --- | --- |
| 200 µg/mL | 110.16±1.96 |
| 100 µg/mL | 102.89±4.82 |
| 50 µg/mL | 103.25±2.01 |
| 25 µg/mL | 99.64±1.25 |
| 12.5 µg/mL | 108.25±1.06 |

Optical density was measured for *S. aureus* cultures incubated with different concentrations of vitellogenin (Vg). Relative growth was calculated in relation to the bacterial control without Vg.

**References**

Benrabaa S, Orchard I, Lange AB. A critical role for ecdysone response genes in regulating egg production in adult female Rhodnius prolixus. PLoS One. 2023;18e0283286.

De Brito TF, Coelho VL, Cardoso MA, De Abreu Brito IA, Berni MA, Zenk FL, et al. Transovarial transmission of a core virome in the Chagas disease vector Rhodnius prolixus. PLoS Pathog. 2021;17. https://doi.org/10.1371/journal.ppat.1009780.

Faria-Reis A, Santos-Araújo S, Pereira J, Rios T, Majerowicz D, Gondim KC, Ramos I. Silencing of the 20S proteasomal subunit-α6 triggers full oogenesis arrest and increased mRNA levels of the selective autophagy adaptor protein p62/SQSTM1 in the ovary of the vector Rhodnius prolixus. PLoS Negl Trop Dis. 2023;Jun 2;17(6):e0011380. https://doi.org/10.1371/journal.pntd.0011380

Guizzo MG, Parizi LF, Nunes RD, Schama R, Albano RM, Tirloni L, Oldiges DP, Vieira RP, Oliveira WHC, Leite MS, Gonzales SA, Farber M, Martins O, Vaz IDS Jr, Oliveira PL. A Coxiella mutualist symbiont is essential to the development of Rhipicephalus microplus. Sci Rep. Dec. 2017;14;7(1):17554. https://doi.org/10.1038/s41598-017-17309-x

Leyria J, Orchard I, Lange AB. Impact of JH Signaling on Reproductive Physiology of the Classical Insect Model, Rhodnius prolixus. Int. J. Mol. Sci. 2022;23. https://doi.org/10.3390/ijms232213832.

Majerowicz D, Alves-Bezerra M, Logullo R, Fonseca-De-Souza AL, Meyer-Fernandes JR, Braz GRC, et al. Looking for reference genes for real-time quantitative PCR experiments in Rhodnius prolixus (Hemiptera: Reduviidae). Insect Mol Biol. 2011;20: 713–722. https://doi.org/10.1111/j.1365-2583.2011.01101.x

Pereira J, Rios T, Amorim J, Faria-Reis A, de Almeida E, Neves M, et al. Functional characterization of vitellogenin unveils novel roles in RHBP uptake and lifespan regulation in the insect vector Rhodnius prolixus. Insect Biochem Mol Biol. 2025;180:104301.

Ursic-Bedoya R, Buchhop J, Joy JB, Durvasula R, Lowenberger C. Prolixicin: a novel antimicrobial peptide isolated from Rhodnius prolixus with differential activity against bacteria and Trypanosoma cruzi. Insect Mol. Biol. 2011;20, 775–786. https://doi.org/10.1111/j.1365-2583.2011.01107.x

Vieira CS, Waniek PJ, Castro DP, Mattos DP, Moreira OC, Azambuja P. Impact of Trypanosoma cruzi on antimicrobial peptide gene expression and activity in the fat body and midgut of Rhodnius prolixus. Parasit. Vectors. 2016;9, 119. https://doi.org/10.1186/s13071-016-1398-4
